# A Graphene Acid - TiO_2_ Nanohybrid as Multifunctional Heterogeneous Photocatalyst for the Synthesis of 1,3,4-Oxadiazoles

**DOI:** 10.1021/acsami.2c07880

**Published:** 2022-07-25

**Authors:** Martina Sciarretta, Mariam Barawi, Cristina Navío, Víctor A. de la Peña O’ Shea, Matías Blanco, José Alemán

**Affiliations:** † Organic Chemistry Department, Universidad Autónoma de Madrid, Madrid 28049, Spain; ‡ Department of Pharmacy, University of Naples “Federico II” (UNINA), Naples I-80131, Italy; § Photoactivated Processes Unit, IMDEA Energy, Avda. Ramón de la Sagra, 3, Móstoles, Madrid 28935 Spain; ∥ IMDEA Nanociencia, Ciudad Universitaria de Cantoblanco, c/Faraday 9, Madrid 28049, Spain; ⊥ Institute for Advanced Research in Chemical Sciences (IAdChem), Universidad Autónoma de Madrid, Madrid 28049, Spain; # Center for Innovation in Advanced Chemistry (ORFEO−CINQA), Universidad Autónoma de Madrid. Madrid 28049, Spain

**Keywords:** photocatalysis, graphene acid, TiO_2_, oxadiazoles, electronic communication

## Abstract

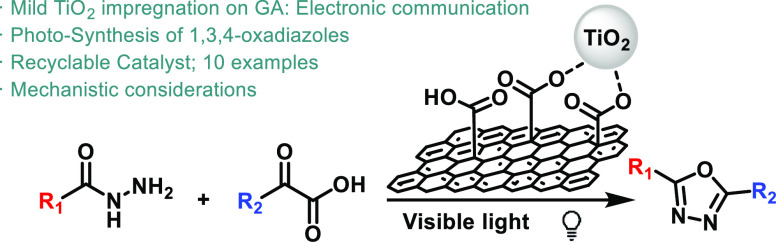

The immobilization of TiO_2_ nanoparticles on graphene acid (GA), a conductive graphene derivative densely functionalized with COOH groups, is presented. The interaction between the carboxyl groups of the surface and the titanium precursor leads to a controlled TiO_2_ heterogenization on the nanosheet according to microscopic and spectroscopic characterizations. Electronic communication shared among graphene and semiconductor nanoparticles shifts the hybrid material optical features toward less energetic radiation but maintaining the conductivity. Therefore, GA-TiO_2_ is employed as heterogeneous photocatalyst for the synthesis of 2,5-disubstituted 1,3,4-oxadiazoles using ketoacids and hydrazides as substrates. The material presented enhanced photoactivity compared to bare TiO_2_, being able to yield a large structural variety of oxadiazoles in reaction times as fast as 1 h with full recyclability and stability. The carbocatalytic character of GA is the responsible for the substrates condensation and the GA-TiO_2_ light interaction ability is able to photocatalyze the cyclization to the final 1,3,4-oxadiazoles, demonstrating the optimal performance of this multifunctional photocatalytic material.

## 1 Introduction

**Scheme 1:**
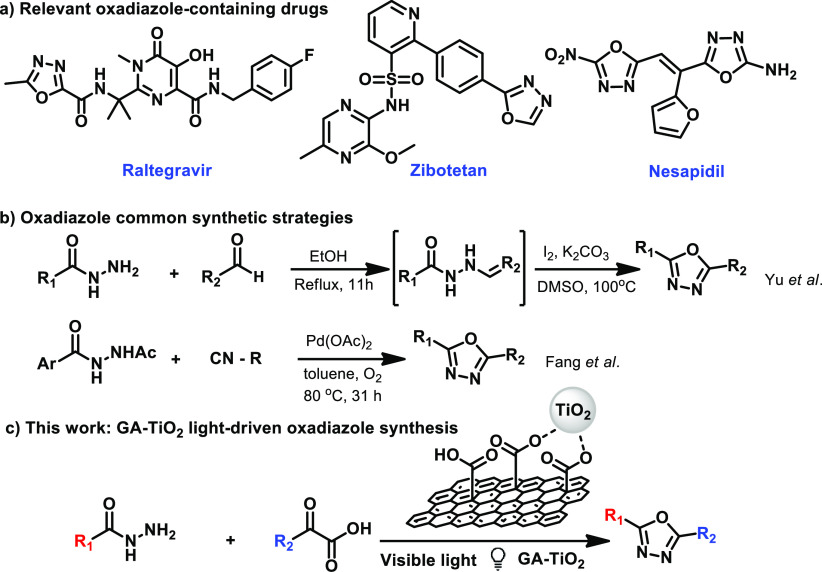
(a) Biologically Active Molecules Containing 1,3,4-Oxadiazole Motifs. (b) Representative Approaches to Obtain 1,3,4-Oxadiazoles Compared to (c) GA-TiO_2_ Nanohybrid Catalyzed Synthesis of 2,5-Disubstituited 1,3,4-Oxadiazoles on This Work

*N*-containing heterocycles, in particular five-membered rings, are of particular relevancy since they are often included in biologically active molecules skeletons,^1^ and this motif is usually employed in medicinal chemistry too.^2^ Among them, 1,3,4-oxadiazoles are privileged scaffolds present in the structure of many marketed and clinically used drugs, such as Raltegravir, Zibotentan, or Nesapidil, which show important biological properties such as antiretroviral, anticancer, or antihypertensive activity (Scheme 1a).^3^ Nevertheless, 1,3,4-oxadiazoles have also been demonstrated to possess antifungal, antibacterial, antioxidant, or anti-inflammatory properties.^4,5^ Their application in the development of advanced light-sensitive electroluminescence and photocatalytic materials has also been explored.^6,7^ Therefore, it is quite desirable to develop and/or improve novel and scalable synthetic routes currently available to yield this important class of molecules. Among them, high-temperature condensation reactions,^8,9^ which can be metal-catalyzed,^10,11^ are the preferred methods for their synthesis (Scheme 1b). However, the photocatalytic synthesis of these heterocyclic scaffolds is still a very challenging topic, opening up the possibility of carrying out more sustainable organic synthesis.

In this sense, TiO_2_ is very abundant in Earth crust and has been recognized as an eco-friendly reagent that presents high photostability and chemical inertness.^12,13^ Thus, TiO_2_ owns widespread application as photocatalyst, from water splitting to pollutants removal and also in organic synthesis.^14−16^ Nevertheless, some disadvantages arise for the general TiO_2_ employment. For instance, it presents a huge electron–hole recombination rate after the excitation process which is usually faster than the interaction with the target reactant,^17^ thus justifying the previously discussed moderate behavior. In addition, the large TiO_2_ band gap energy limits its absorption to the deep UV,^18^ which is a very aggressive radiation for sensitive organic molecules. Furthermore, a high capability of reactive oxygen species generation, able to destroy organic material, is present in the TiO_2_-mediated photophenomena.^19^ Therefore, bare TiO_2_ owns limitations that humpers its general application as synthetic tool. In order to avoid these drawbacks and covert TiO_2_ into a more efficient photocatalyst, heterogenization on an adequate support is a recurrent solution.^20^ Thence, several reports dealt with the nanocomposite synthesis based on graphene derivatives and TiO_2_ for a wide gamut of applications.^21^ Within this immobilization, the carbon nanomaterial interacts with the band structure of the semiconductor,^22^ resulting in a bathochromic shift allowing the chemistry to be played with visible radiation.^23^ This electronic communication entails conductive platforms, thus preventing the insulator graphene oxide to satisfy the general mechanism and requiring further reducing steps that could result in detrimental stabilization effects for the TiO_2_.^24^

Very recently, a new graphene derivative called graphene acid (GA) came on the graphene derivatives scene.^25^ GA consists, thanks to synthetic design, in a conductive graphene sheet (the C sp^2^ content is higher than 70%) densely and specifically functionalized with COOH groups (up to 11%).^26^ GA has been employed as carbocatalyst itself in the synthesis of aldehydes^27^ or has been combined with homogeneous species for catalytic hybrid nanomaterial’s construction both with organometallic units and nanoparticles.^28,29^ Indeed, the COOH groups are very prompted for functionalization using the amide chemistry, sustaining controlled nanoparticle growing or conferring GA its solid acid character. In addition, GA has shown excellent performance clearly superior to homogeneous counterparts and the benchmark GO analogous as a result of this particular set of properties.^30^ Regarding the photocatalytic application, GA has been employed for the photochemical reduction of CO_2_.^31^ Hence, GA was concluded to interact with the light and assist the photoactive unit at the CO_2_ reduction following a different pathway compared to the homogeneous counterpart. Nevertheless, GA has been never combined, to the best of our knowledge, with inorganic semiconductors such as TiO_2_ aiming photocatalytic procedures. In addition, the synthetic application of GA is still very limited.^30^ In this regard, a light-mediated synthesis of 1,3,4-oxadiazoles could be planned with this especial hybrid taking advantage of both the carbocatalytic character of the material and the photoactivity of an immobilized TiO_2_ nanoparticle.

In this work, we report the functionalization of GA with TiO_2_ clusters by noncovalent interactions via the in situ hydrolysis of a titanium alkoxide. As a result, TiO_2_ clusters of 4 nm in mean diameter were found populating the material’s sheets, as demonstrated by the microscopic and spectroscopic characterization performed. The hybrid material was found active in the light-driven synthesis of 1,3,4-oxadiazoles bearing aromatic and aliphatic wingtips upon reaction between ketoacids and hydrazides. Compared to bare TiO_2_, the hybrid material presents enhanced activity thanks to the combination of the carbocatalytic character, that yields an imine to be furtherly cycled in a light-driven reaction activated by the photosensitive unit. The electronic communication between graphene and TiO_2_ facilitates this second process of the cascade. High stability, recyclability, and structural variety in the yielded products characterize this heterogeneous catalyst too.

## 2 Results and Discussion

**Scheme 2:**
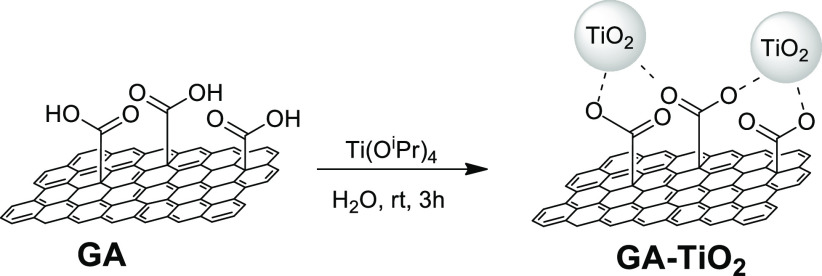
Synthesis of GA-TiO_2_ from Titanium Alkoxide

The GA material was prepared according to a previously described protocol based on exfoliation and reaction of fluorographene with a cyanide source followed by further hydrolysis.^25^ The procedure yielded a black powder that consists of a few-layered graphene scaffold with a typical lateral extension of 400–900 nm and also densely functionalized with COOH groups (see Supporting Information (SI) for further characterization details). In particular, the C sp^2^ peak dominated the C 1s XPS core level region, and the band corresponding to COO bonds (288.4 eV of binding energy) was observed as the most intense of the surface oxygen chemistry (see below). Thus, the titanium alkoxide precursor was adjusted as a function of the characteristics of the graphene platform. Hence, a stoichiometric loading of Ti(O^i^Pr)_4_ vs the quantity of COOH groups was set to be hydrolyzed in water in the presence of GA to yield the nanocomposite GA-TiO_2_ (see Scheme 2 and SI for further experimental details). It is worthy to highlight that any type of annealing procedure or even a heating treatment was performed during the titanium immobilization at the surface of the graphene material.^32^

**Figure 1:**
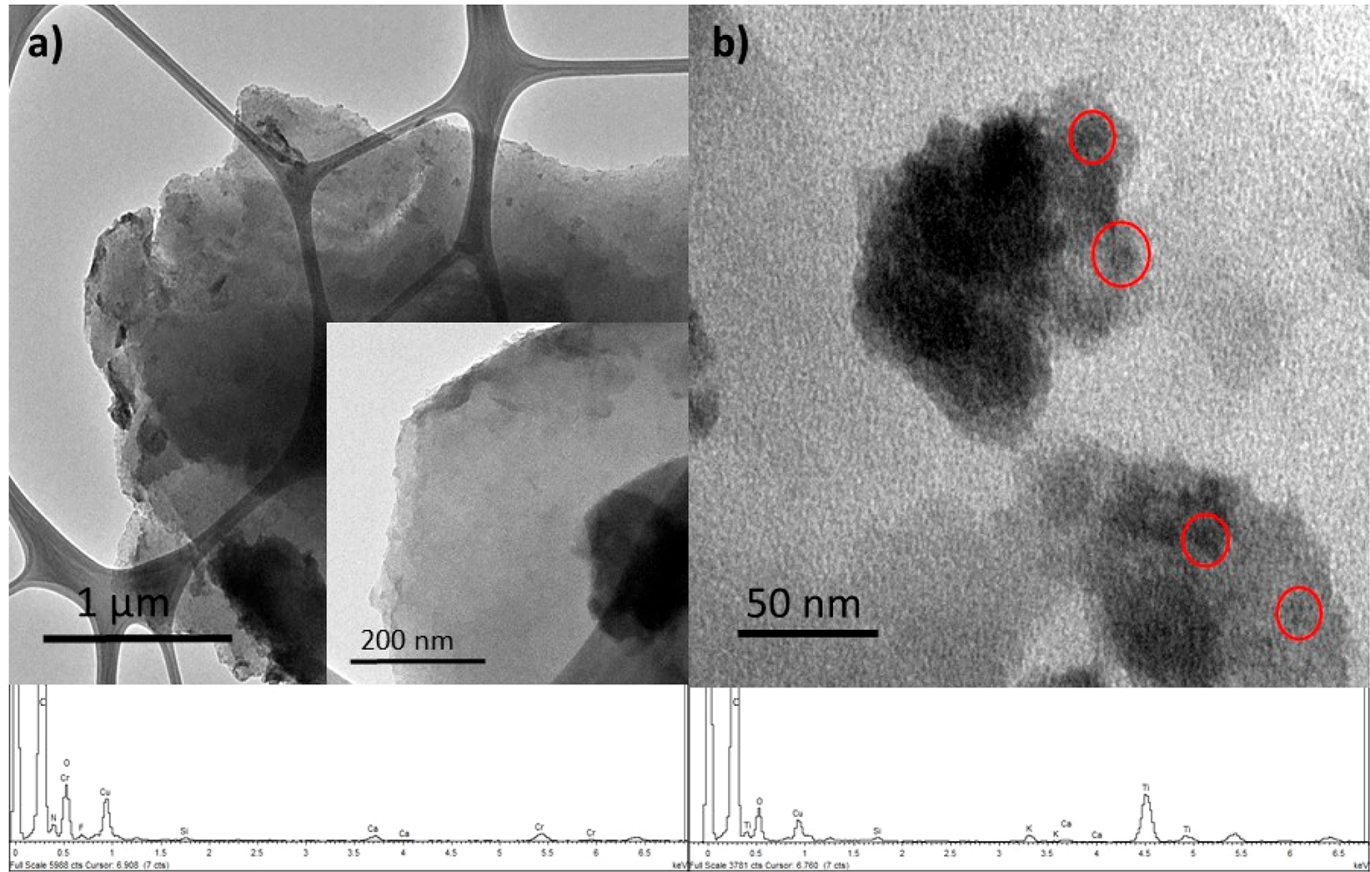
TEM images of (a) pristine GA (inset is a high magnification image) and (b) hybrid material GA-TiO_2_ (red circles highlight TiO_2_ nanoparticles). Lower panels depict the corresponding EDX spectra.

Initially, we hypothesized that the COOH groups could sustain the hydrolysis of the titanium alkoxide. Indeed, this particular surface functional group was previously demonstrated to present affinity for metal species and confer the solid acid character to GA.^29,33^ This behavior could be employed for anchoring TiO_2_ nanoparticles at the surface by noncovalent interactions. Indeed, TEM observations of the hybrid material after the titanium introduction showed clusters with inorganic appearance distributed over graphene sheets (Figure 1). Those TEM images indicate that the hydrolysis of the titanium alkoxide precursor was produced only at the surface of the graphene derivative as all the inorganic material was deposited over the graphene nanosheets. Deeper observations revealed that those inorganic clusters were composed of individual sphere-like nanoparticles with a size around 4 nm (see SI Figure S14 for further details). In addition, the clusters were mainly placed at the basal plane of the graphene sheet (i.e., the population of the sheet’s edge was very low), which agrees with the inherent location of the COOH groups designed by the performed synthetic protocol. Those nanoparticles were identified as titanium species by EDX analysis. Thus, the morphological aspect of the GA-TiO_2_ sample totally matches previous reports dealing with graphene oxide-TiO_2_ nanocomposites.^34−36^ However, the special properties of this GA in terms of electron conductivity with dense and controlled oxygen functionalization allowed a good nanoparticles hosting just by simple room-temperature wet-impregnation reaction with the titanium precursor.

**Figure 2:**
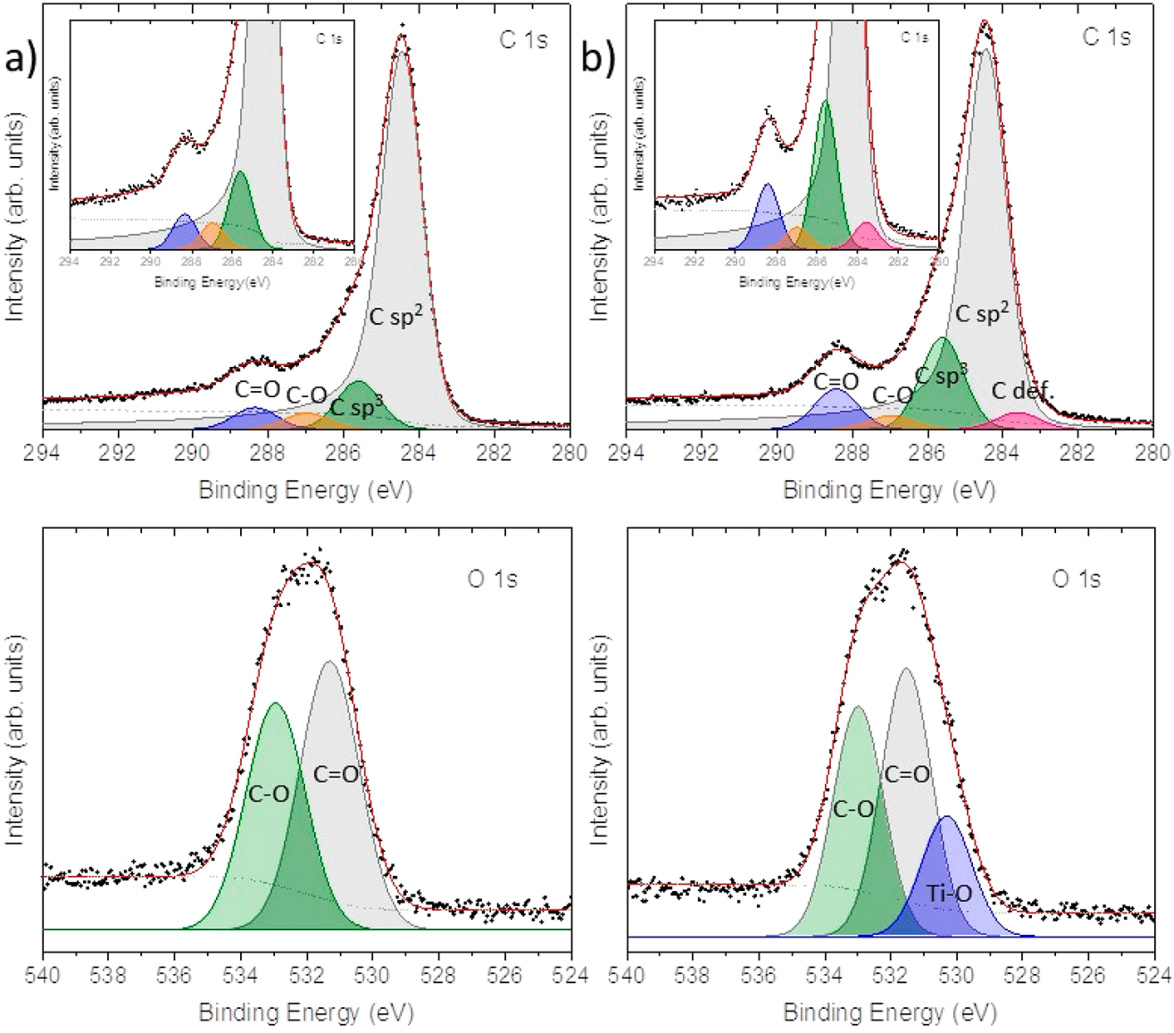
XPS analysis of samples (a) GA and (b) GA-TiO_2_ (upper panels: XPS C 1s core level region, and the inset represents a zoom over the region of interest; lower panels: XPS O 1s core level region).

Elemental analysis agrees with the introduction of TiO_2_ at the surface of GA. Indeed, the oxygen content was increased from 28.1% wt. to 37.2% wt. (see SI Table S1). Since the titanium impregnation reaction over GA was conducted at room temperature, the hydrolysis of the titanium alkoxide precursor to give TiO_2_ must be at the surface of GA.^37^ In addition, TXRF measurements of the sample determined a Ti loading of 4.4% in the sample GA-TiO_2_, which totally matches the elemental analysis determination (see SI Table S2 and Figure S15). Nevertheless, the chemical composition of the oxide was studied by means of XPS. Hence, C 1s, O 1s, and Ti 2p XPS core level regions came easily across in the XPS survey spectrum of sample GA-TiO_2_ (see SI Figure S16), agreeing with the TiO_2_ introduction at the graphene material. High resolution spectra shed more light among the different chemical species present at the sample’s surface. Indeed, the C 1s XPs core level region of the TiO_2_ impregnated graphene sample GA-TiO_2_ is almost identical to the pristine GA with the exception of the COO bonds (Figure 2 upper panels), which is fitted as a broader band compared to the pristine GA spectral features.^24,25^ This fact suggests that only the carboxylic acids of the GA nanosheet is the responsible for the allocation of the TiO_2_ nanoparticles. Nevertheless, the similarity on the rest of the C 1s XPS core level spectrum, despite the emergence of defects peaks at 283.6 eV of BE as a result of the TiO_2_ immobilization,^38^ might indicate that neither any bond nor new interactions with the basal plane of the graphene material have been created between GA and the titanium precursor. Indeed, the Ti 2p XPS core level region appeared as a doublet whose Ti 2p_3/2_ peak was located at 458.8 eV of binding energy (BE) (see SI Figure S17),^39^ matching the chemical shift of TiO_2_ species. In agreement with C 1s analysis, any component that could be assigned as an eventual Ti–C bond could be found. Finally, the O 1s XPS core level region also agreed with the previously discussed data (Figure 2 lower panels). A new component with respect to pristine GA merged out in the sample GA-TiO_2_ at around 530 eV of BE,^40^ which might be related to the new C–O–Ti bond formed through the carboxylic acids in the semiconductor impregnation reaction. As a whole, the XPS analysis supports that the carboxylic acids of the material had intervened in the hydrolysis of the Ti(O^i^Pr)_4_, allocating the resulting TiO_2_ as a form of nanoparticles only at the COOH groups.

**Figure 3:**
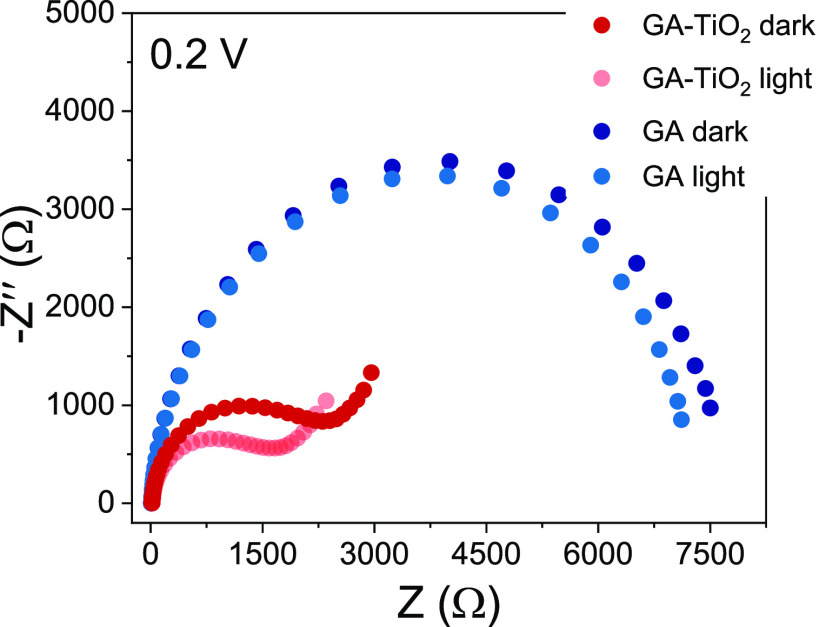
Nyquist plots obtained by electrochemical impedance spectroscopy (EIS) under dark and illumination conditions at 0.2 V vs Ag/AgCl in 0.2 M aqueous Na_2_CO_3_ of GA and GA-TiO_2_ samples.

The interaction of the samples pristine GA and functionalized GA-TiO_2_ with the light was studied by means of UV–vis spectroscopy using stable colloidal suspensions. GA presented an almost plain absorption spectrum in the whole visible range, with two very weak absorption shoulders at around 260 and 230 nm (see SI Figure S18a). Conversely, GA-TiO_2_ showed an absorption maximum at 280 nm of wavelength as a result of the interaction of the TiO_2_ with the light. Even this GA has never been combined before with TiO_2_-type semiconductors, the GA-TiO_2_ optical features resembled very similar to the typical graphene oxide–TiO_2_ nanocomposites (and different from bare TiO_2_, see SI Figure S19),^41,42^ highlighting the established electronic communication among both partners. In addition, the UV–vis emission spectrum in the 300–700 nm range for sample GA-TiO_2_ displayed a maximum related to the C=C bonds lying deep in the UV (315 nm), accompanied by a clear shoulder between 400 and 500 nm that is not observed in the pristine GA optical behavior. The hybrid’s emission of radiation also lies in the typical range of graphene oxide-TiO_2_ materials according to different literature reports,^43−45^ leading to the absorption of a higher portion of the visible spectrum. In agreement with this set of observations, the ability of the hybrid material GA-TiO_2_ to absorb light and transfer charge at the solution interface was found quite different compared to the typical behavior of bare TiO_2_. To study the ability of the materials to charge transfer toward a solution, electrochemical impedance spectroscopic (EIS) measurements were performed at 0.2 V vs Ag/AgCl (inside the stability window of the material, see SI Figure S20) under dark and simulated solar illumination.^46−48,49^Figure 3 exhibits the Nyquist plots obtained for both pristine GA and GA-TiO_2_ samples. Interestingly, under dark conditions both systems presented great conductivity, but the hybrid material GA-TiO_2_ showed almost an improved conductive character by more than twice (3000 vs 7500 Ω). Indeed, pristine GA was previously characterized as a very conductive graphene derivative,^25,26^ and this particular material showed a charge transfer resistance which is in agreement with previous works.^29^ However, there is a slight conductivity enhancement for both samples when the solar light simulator was used, and this difference becomes more relevant in the case of GA-TiO_2_. In the GA situation, this fact can be attributed to the small semiconductor character as already demonstrated previously by first principle calculations (band gap energy ∼0.5 eV),^25^ but the observed conductivity difference for GA-TiO_2_ is higher compared to pristine GA and also to bare TiO_2_ (see SI Figure S20c). Indeed, GA-TiO_2_ is the most conductive sample under solar light simulator illumination in this study, evidencing an eventual activity of the TiO_2_ nanoparticles on the GA network as photocatalyst combined with a very efficient charge transfer ability toward the electrolyte.

Having in hand this material based on graphene acid nanosheets with immobilized TiO_2_ nanoparticles able to interact with light, we wanted to study its ability as photocatalyst in the synthesis of biological relevant 1,3,4-oxadiazoles. As a model reaction, as depicted in Table 1 (entry 1), GA-TiO_2_ was able to obtain 2,5-diphenyl-1,3,4-oxadiazole **3a** with a conversion higher than 98% (determined by NMR) in an overnight reaction using white LED as illumination source.^16^ Reaction conditions were optimized in order to achieve the best performance possible (see SI for details). Thus, different bases were employed, such as tertiary amines (triethylamine or di-isopropylethylamine) or inorganic bases (NaOH or K_2_CO_3_), yielding product **3a** with a maximun conversion of 65% (Table 1 entry 2). However, the best result was obtained using 2 equiv of Na_2_CO_3_ as base (conversion higher than 98%). Different solvents were also screened, obtaining the best result in water (conversion >98%), meanwhile *N*,*N*-dimethylformamide (DMF), methanol (MeOH) or acetonitrile (MeCN) also afforded lower conversions of **3a** (Table 1 entry 3). The source of light was also very important, only detecting appreciable levels of conversion using a white LED, whereas the use of blue or green visible light provoked lower conversions of the oxadiazole **3a** (Table 1 entry 4). The oxadiazole was not observed without any source of light or without the addition of the photocatalyst GA-TiO_2_ (Table 1 entry 5). Interestingly, a reaction performed under inert atmosphere did not afford the formation of the oxadiazole **3a**, highlighting the role of molecular oxygen in the mechanism of this light-driven transformation. Finally, the reaction can be scaled up to the 2 mmol scale with a very impressive 97% conversion.

**Table 1:** Optimization of the Diphenyl-1,3,4-oxadiazole **3a** Synthesis Catalysed by GA-TiO_2_.

entry^a^	variation from standard conditions	conversion (%)^b^
1	none	>98
2	base (none, amine or inorganic)	0–65
3	solvent (DMF, DMSO, MeOH, MeCN)	0–62
4	light (dark, 360 nm, 385 nm, blue or green)	0–38
5	no catalyst	0
6	inert atmosphere	0
7	2 mmol scale	97

a Reaction conditions: 0.1 mmol of substrates, 1 mg of catalyst, 1 mL of water, white LED illumination, air atmosphere, 16 h, room temperature.

b Determined by ^1^H NMR.

**Figure 4:**
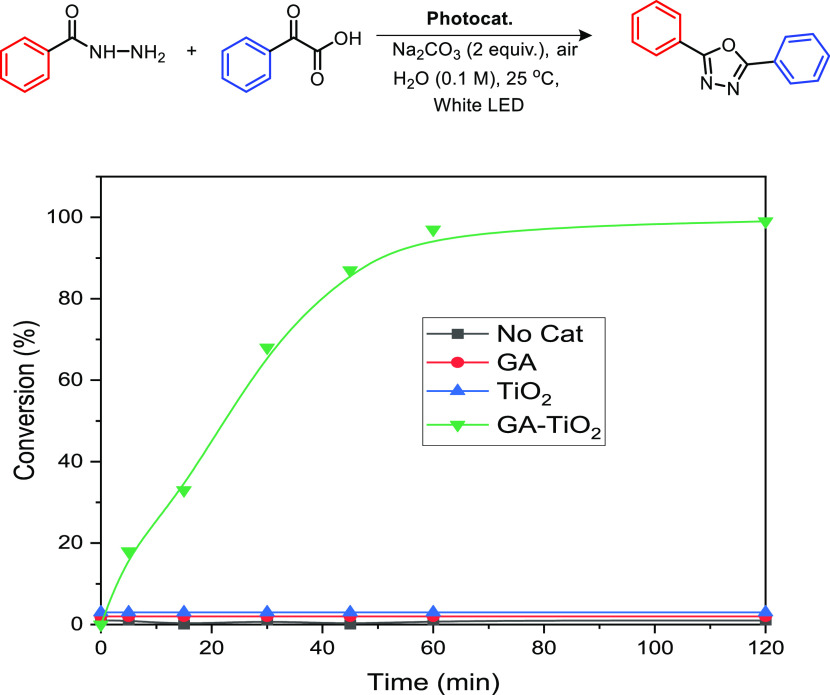
Temporal evolution in the formation of oxadiazole **3a** catalyzed by different samples under study.

Once we optimized the best reaction conditions, we also studied the kinetics of the diphenyl-1,3,4-oxadiazole formation. Under the parameters depicted in Table 1, GA-TiO_2_ presented a pseudo-first order reaction kinetics (Figure 4). Upon turning on the light, the conversion started to rise without any kind of induction period and the material generated **3a** with almost full conversion (97%) in just 1 h of irradiation. Elongating the reaction time did not modify the outcome of the reaction, highlighting the stability of the generated product **3a**. With this outstanding performance, a maximum turnover number (TON) of 97 was calculated for GA-TiO_2_ photocatalyst under the standard conditions, with an initial turnover frequency (TOF_0_) calculated after 5 min of reaction of 217 h^–1^. Nevertheless, the scaled-up reaction accounted a maximum TON of 1940, which makes this material comparable or better to other state-of-the-art (photo)catalysts (including noble metals) for the synthesis of this family of molecules (see SI Table S6).

We also compared the performance in the light-driven synthesis of **3a** with pristine GA as photocatalyst of the transformation (red-line, Figure 4). Without any photoactive center in the structure, pristine GA, as expected, was unable to yield **3a**. Nevertheless, GA was not innocent at all, and after analysis of the crude of this particular control reaction, we did not detect neither the presence of the ketoacid nor the hydrazide. Instead, GA was able to carbocatalyse the synthesis of the imine **4a** resulting from the condensation of the starting reactants thanks to the inherent solid acid character (see SI Scheme S1). Other oxidized carbon nanomaterials reported in the literature also presented this carbocatalytic behavior.^26,50−56^ The surface chemistry is still active in the sample **GA-TiO**_**2**_ too. Indeed, GA-TiO_2_ under dark conditions could afford the intermediate imine **4a**. Thus, the heterogenization of the semiconductor seemed to not block the oxygen carbocatalytic active sites, matching the XPS analysis. This situation was completely different at the noncatalyzed reaction, where the mixture of hydrazide and ketoacid was found condensed to the imine **4a** only in a very low 8% conversion, with obviously negligible reactivity toward the cycled product **3a**, as depicted in Table 1 too. We also wanted to compare the behavior of our graphene-based catalyst with a nonhybrid catalytic material reference. To perform this task, a commercial TiO_2_ (Degussa P25) sample was employed. In order to replicate the semiconductor loading on the heterogenized material GA-TiO_2_ according to the TXRF analysis, 1 μmol of P25 was employed. This experiment without the hybrid material resulted in a negligible formation of the oxadiazole **3a** starting from the ketoacid and the hydrazide using the standard conditions of Table 1. Indeed, the mixture was found unaltered after 2 h of illumination.

**Scheme 3:**
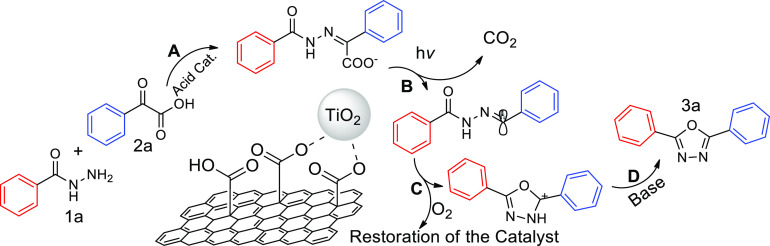
Reaction Pathway for the Synthesis of Oxadiazole **3a** Catalysed by GA-TiO_2_

With all the gathered data, we propose the mechanistic pathway depicted in Scheme 3. According to the experiments discussed above, the material itself is able to promote the intermolecular condensation reaction **A** between **1a** and **2a**, yielding an imine prompted to undergo a light-driven decarboxylation reaction **B**. As a result, a radical is generated which owns the appropriate geometry to be cycled with the assistance of the supported photoactive unit TiO_2_ in the intramolecular cyclization reaction **C**. Finally, the base is responsible of H abstraction in the reaction **D** to yield the final 1,3,4-oxadiazole. Since the reaction performed under inert atmosphere resulted in negligible conversion, the molecular oxygen must be the responsible of the restoration of the photocatalyst for the further catalytic turnover.^57−59^

Additional control experiments were run in order to support the proposed mechanistic pathway. Indeed, a reaction performed with 1 μmol of Degussa P25 TiO_2_ as photocatalyst using as substrate the imine **4a** yielded the oxadiazole **3a** with a 47% conversion after 16 h irradiation (see SI Scheme S2). This experiment demonstrated that the photoactive unit is only able to yield the heterocycle **3a** in moderate conversion when the imine **4a** is already preformed, highlighting the bifunctional character of our material as acid Lewis catalyst for the condensation and photocatalyst for the cyclization, as well as enhancing its photocatalytic performance.^60,61^ Therefore, the heterogenization over GA becomes a very smart solution to avoid additional synthetic steps, since this GA material not only enhances the performance of the overall system (comparing homogeneous and heterogenized runs), but also prepares and sorts the required substrate to the photoactive unit.^62^

After analysis of the reaction pathway that GA-TiO_2_ employs to yield the 1,3,4-oxadiazoles, we studied the scope of the catalyst in a general synthesis of this family of important heterocycles. The exploration of the reaction scope is depicted in Table 2 with reaction time selected to be 3 h in order to ensure high conversion for all the substrates analyzed. Thus, 2,5-diphenyl-1,3,4-oxadiazole **3a** was obtained in a 74% isolated yield. Other aromatic residues, such as the symmetric ditolyl-oxadiazole **3b** was synthesized in a 73% yield starting from the *p*-tolyl ketoacid and hydrazide. Asymmetric oxadiaxoles with two different substituents in five-membered-ring was also synthesized by GA-TiO_2_. For instance, an isopropyl or a methyl combined with a phenyl ring could be obtained (products **3c**, 83% yield and **3d**, 72% yield) with a very good performance using the corresponding aliphatic ketoacids with phenyl hydrazide. We could also obtain oxadiazole **3d** using the other combination of reactants, that is, phenyl ketoacid and acetyl hydrazide. However, this reaction afforded product oxadiazole **3d** in 69% yield. Other asymmetric 1,3,4-oxadiazoles combining aliphatic and aromatic arms could be synthesized such as the combination of *p*-tolyl-methyl **3e** (80% yield). Furthermore, the reaction is tolerant to halides in the aromatic ring (*p*-chlorophenyl-methyl **3f** (70% yield) and *p*-bromophenyl-methyl **3g** (78% yield) configurations), achieving the target products with good performance. The presence of electron withdrawing groups (EWG) or electron donor groups (EDG) did not modify the outcome of the reaction, since the oxadiazole **3h** with a CF_3_ group in the aromatic ring or the oxadiazole **3i** bearing a methoxy group in the phenyl ring were obtained with very similar performance (88% yield for **3h** and 78% yield for **3i**). Lastly, a fully aliphatic oxadiazole **3j** bearing isopropyl and methyl groups was synthesized in a very good 74% yield. As a whole, GA-TiO_2_ is presented as a versatile catalyst able to yield oxadiazoles with important structural variety.

**Table 2:**
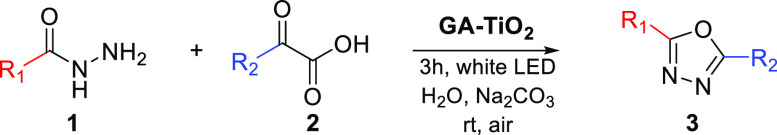

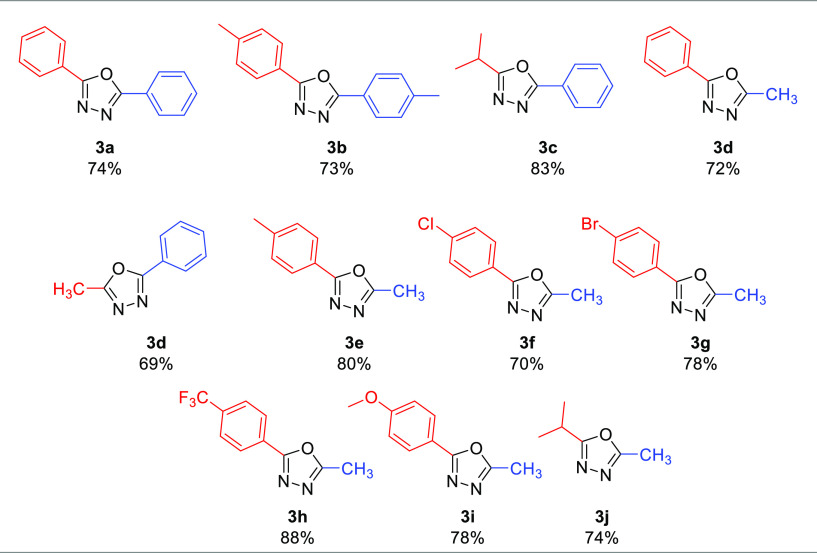
Scope of the Photocatalyst in the Synthesis of Oxadiazoles.^a^

a Reaction conditions: 0.1 mmol of substrates, 2 equiv of Na_2_CO_3_, 1 mL of H_2_O, 1 mg of GA-TiO_2_ as catalyst, air atmosphere, white LED illumination for 3h. Values stand for isolated yield.

**Figure 5:**
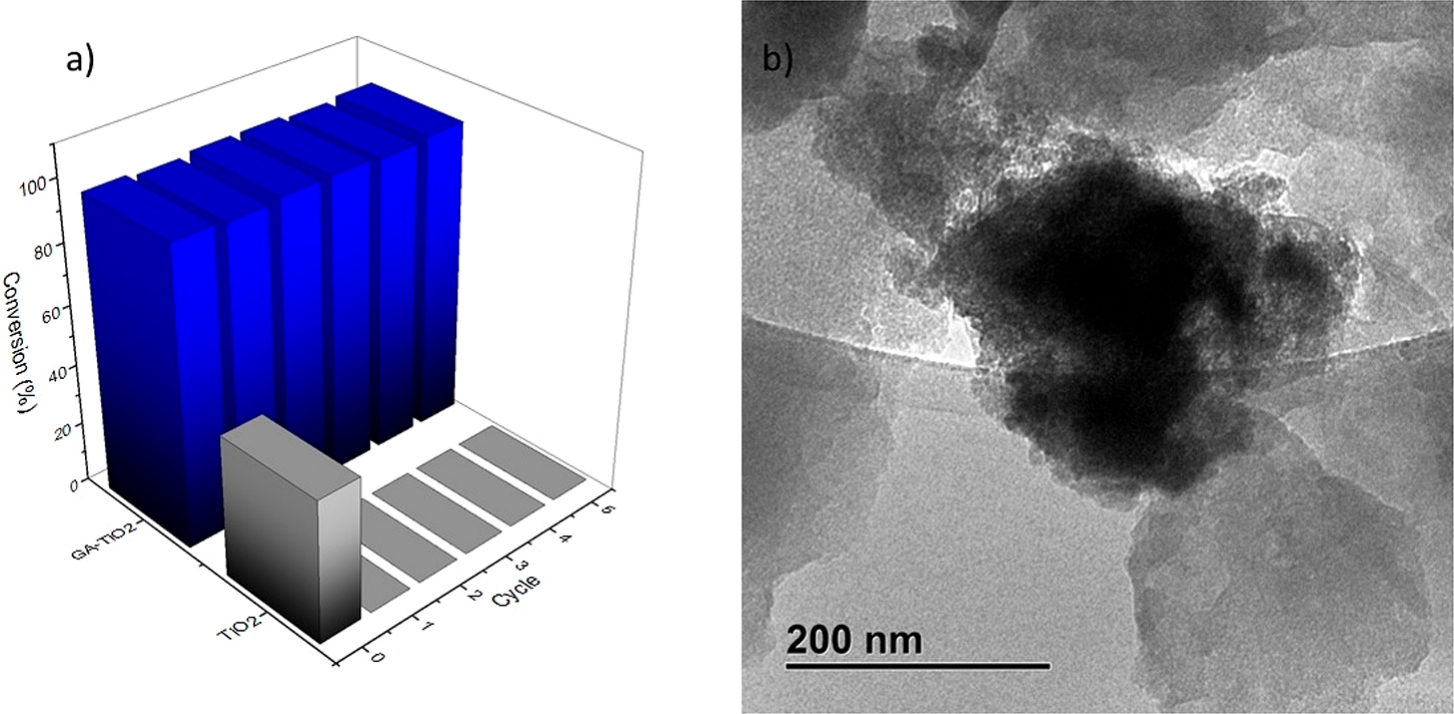
(a) Cycling study in the synthesis of **3a** by GA-TiO_2_ and TiO_2_. (b) TEM image of the recovered GA-TiO_2_ after the catalytic reactions.

Finally, the ability of our heterogeneous photocatalyst of being recovered combined with its stability during operation was evaluated. Hence, once the model reaction for the synthesis of diphenyl-1,3,4-oxadiazole **3a** was completed, the black powder consisting of GA-TiO_2_ was separated from the reaction medium by means of filtration through a polypropylene membrane, profusely washed with a mixture of water and organic solvents for the removal of all the impurities and submitting the clean solid to further reaction cycles by adding fresh reactants but without adding in any case fresh catalyst. Interestingly, GA-TiO_2_ maintained very good levels of conversion above 96% of **3a** after five recovery cycles (Figure 5a), while bare TiO_2_ was not able to be recycled after the very first fresh reaction even employing as substrate the precondensed imine-type molecule **4a** described above. In addition, we also analyzed the reaction mother liquids in order to find if any titanium species were leached out during the catalytic process. For our delight, the mother liquids contained levels of titanium below 0.3 ppm according to TXRF analysis (see SI Table S7 and Figure S23. Furthermore, the recovered material GA-TiO_2_ presented a very similar morphological composition compared to the fresh one not used for a catalytic experiment since the TiO_2_ clusters did not appear to experience any growing after the reaction (Figure 5b). In addition, the elemental and TXRF analysis, combined with the XPS surveying of the recovered material did not show any modification compared to the fresh one (see SI Figures S21 and S22 and Table S3).

## 3 Conclusions

This work describes the heterogenization of TiO_2_ on graphene acid, a conductive and oxidized graphene derivative which contains, as main oxygen functional group, a large amount of carboxylic acids. These carboxylic acids are the responsible of hydrolyzing the titanium precursor and host the in situ formed 4 nm wide TiO_2_ nanoparticles by noncovalent interactions at the basal plane of the graphene nanosheet. In addition, electronic communication was stablished among the inorganic clusters and the graphene material because a bathochromic shift with enhanced charge transfer toward the medium under illumination were observed in the optical features of the hybrid material GA-TiO_2_ compared to pristine GA and bare TiO_2_. Therefore, GA-TiO_2_ is employed as heterogeneous photocatalyst for the visible-light-driven synthesis of 2,5- aliphatic and/or aromatic disubstituted 1,3,4-oxadiazoles using ketoacids and hydrazides as substrates. The hybrid material presented improved photoactivity compared to the commercial and bare TiO_2_ sample, yielding a wide gamut of oxadiazoles with different structural parameters in reaction times as fast as 1 h. Full recyclability over more than five reaction cycles combined with stability in operation without TiO_2_ leaching. The carbocatalytic character of GA is the responsible for the substrates condensation forming imines, and also the further photocatalytic cyclization reaction. Thus, a multifunctional catalytic material is prepared by a rational and simple heterogenization protocol that avoids synthetic steps.

## Supporting Information Available

Experimental section, synthesis, extended characterizations, and catalytic data (PDF)
