# Supplementary material for: A Graphene Acid - TiO2 Nanohybrid as Multifunctional Heterogeneous Photocatalyst for the Synthesis of 1,3,4-Oxadiazoles
Source: ACS Appl Mater Interfaces. 2022 Jul 25;14(30):34975–84. doi: 10.1021/acsami.2c07880 (PMC9827454; doi:10.1021/acsami.2c07880)
Supplement: Supplementary file 1 — am2c07880_si_001.pdf [file am2c07880_si_001.pdf]

Supporting Information

A Graphene Acid - TiO_2 Nanohybrid as Multifunctional Heterogeneous Photocatalyst for the Synthesis of 1,3,4-Oxadiazoles

Martina Sciarretta,[1,2] Mariam Barawi,[3] Cristina Navío,[4] Víctor A. de la Peña O'Shea,[3] Matías Blanco[1] and José Alemán[1,5,6]**

[1] *Organic Chemistry Department, Universidad Autónoma de Madrid, 28049, Madrid, Spain*
[2] *Department of Pharmacy, University of Naples "Federico II" (UNINA), I-80131 Naples, Italy*
[3] *Photoactivated Processes Unit, IMDEA Energy, Avda. Ramón de la Sagra, 3, 28935 Móstoles, Madrid, Spain*
[4] *IMDEA Nanociencia, Ciudad Universitaria de Cantoblanco, c/Faraday 9, 28049, Madrid, Spain*
[5] *Institute for Advanced Research in Chemical Sciences (IAdChem), Universidad Autónoma de Madrid, 28049 Madrid, Spain.*
[6] *Center for Innovation in Advanced Chemistry (ORFEO-CINQA), Universidad Autónoma de Madrid. Madrid-28049, Spain*

* To whom correspondence should be addressed.
Email address: matias.blanco@uam.es jose.aleman@uam.es

Summary

S1. Experimental Section, Synthesis and Characterization of Organic Compounds 2
S2. Supporting materials characterization data 11
S3. Supporting catalytic data 17
S4. Supporting References 21

S1. EXPERIMENTAL SECTION, SYNTHESIS AND CHARACTERIZATION OF ORGANIC COMPOUNDS

General Information, materials and methods

All chemicals, solvents and reagents, including Fluorographite (Merck), were purchased from commercial sources (reagent grade quality or better) and used without further purification, with the exception of 2-oxo-2-(p-tolyl) acetic acid, which was prepared in accordance with a previously reported method,[1] for the synthesis of compound **3b**. Purification of organic products, when necessary, was accomplished by flash chromatography using silica gel (Merck Geduran® Si 60) in an adequate mixture of cyclohexane (CyH) and ethyl acetate (EtOAc) eluents. All the organic products were characterized by comparison of their spectral data with those reported in the literature.

Nuclear Magnetic Resonance (NMR) spectra were acquired on a BRUKER AVANCE spectrometer running at 300 MHz for ^{1}H and are internally referenced to residual solvent signals ($CDCl_3$ referenced at δ 7.26 ppm for ^{1}H NMR, DMSO-d6 referenced at δ 2.50 ppm, CD_3OD referenced at δ 3.34 ppm for ^{1}H-NMR). Data for ^{1}H NMR are reported as follows: chemical shift (δ ppm), multiplicity (s = singlet, bs = broad singlet, d = doublet, dd = doublet of doublets, ddd = doublet of doublet of doublet, dt = doublet of triplets, ddt = doublet of doublet of triplets, t = triplet, sept = septuplet, m = multiplet), coupling constant *J* (Hz) and integration. Transmission Electron Microscopy (TEM) images were acquired with a JEOL-JEM 1400 instrument equipped with a CCD high resolution camera and an Oxford EDX spectrometer *in situ* microprobe. Samples were dropcasted from nanotubes' methanol suspensions on holey-carbon copper grids. For the elemental analysis measurements, a LECO CHNS-932 Analyser (Model NO: 601-800-500) was used. Qualitative and quantitative Total X-Ray Fluorescence analyses (TXRF) were performed with a benchtop S2 PicoFox TXRF spectrometer from Bruker Nano (Germany). TXRF system was equipped with a Mo X-ray source working at 50 kV and 600 μA, a multilayer monochromator with 80% of reflectivity at 17.5 keV (Mo Kα), a XFlash SDD detector with an effective area of 30 mm^2 and an energy resolution better than 150 eV for 5.9 keV (Mn Kα). For deconvolution and integration, commercial Spectra v. 7.5.3 software package from Bruker was used. Fourier Transformed IR (FTIR) were

recorded on a Thermo Nicolet Avatar 380 FT-IR equipped with a Michelson filter interferometer. For the preparation of the samples, 200 mg of dry KBr were mixed with 2 mg of the sample in a mortar. Pressure was then applied with a hydraulic press until a fine pellet was generated. XPS (X-ray Photoelectron Spectroscopy) measurements were performed under Ultra High Vacuum conditions (UHV, with a base pressure of 5×10^{-10} mbar), using a monochromatic Al K_{α} line as exciting photon source for core level analysis (hν = 1486.7 eV). The emitted photoelectrons were collected in a hemispherical energy analyser (SPHERA-U7, pass energy set to 20 eV for the XPS measurements to have a resolution of 0.6 eV) and to compensate the built-up charge on the sample surface it was necessary the use of a Flood Gun (FG-500, Specs), with low energy electrons of 3 eV and 40 µA. C 1s sp^2 centered at 284.4 eV is taken as biding energy reference. Samples were stack on high purity carbon scotch tape on a steel holder and left outgas overnight in high vacuum prior to the measurements. The UV-visible absorption spectroscopy data were acquired using a Cary 50 spectrometer (Varian), in the 200–800 nm range. In this case, powder samples were dispersed in methanol, forming a stable colloidal dispersion. Same suspensions and solutions were employed to record the luminescence spectra, using a JASCO Spectrofluorometer FP-8600 equipped with a TC-815 Peltier thermostated single cell holder (water-cooled) controlled by Spectra Manager Version 2.10.01. HPLC grade methanol solvent and a 10x10 mm light path quartz SUPRASIL® cuvette equipped with a silicone/PTFE septum were used for all measurements. UV-Vis diffuse reflectance spectra (DRS) of the solid powdered samples were obtained by a Perkin Elmer Lambda 1050 UV/Vis/NIR spectrometer and the reflectance spectra were plotted as the tauc plot and the Kubelka-Munk function.

Electrochemical measurements were performed on a standard 3 electrode cell configuration.[2] Current and voltage signals were measured through an Autolab PGSTAT204 potentiostat/galvanostat station equipped with an integrated impedance module FRAII. An ink containing the sample was prepared mixing 2 mg of each material with 15µL of Nafion perfluorinated resin solution (5% wt. in mixture of lower aliphatic alcohols and water, 45%) and 0.5 mL of *N*,*N*-dimethylformamide (DMF).[3] After that, this ink was dropwise deposited and dried on the platinum working electrode. A platinum wire was used as the counter electrode and an Ag wire electrode as the pseudo-reference (calibrated with ferrocene) was also employed. 0.1 M of $[(^{n}Bu)_4)N]ClO_4$ in

dichloromethane (DCM) was utilized as electrolyte. In order to determine the sample's valence and conduction bands energy levels (Vacuum, eV), the oxidation and reduction peaks values obtained in the cyclic voltammetry were corrected by the use of ferrocene redox couple. A modulation amplitude of 10 mV was used in the frequency range from 0,1 Hz to 10,000 Hz in the EIS measurements with a Xe lamp (300 W) equipped with a filter to simulate the solar spectrum.[4] For this set of measurements, samples were dropcasted on indium tin oxide (ITO) coated glass slices as working electrode and a 0.2 M Na_2CO_3 aqueous solution was employed as electrolyte referred to Ag/AgCl.

General protocol for the synthesis of GA

GA was yielded applying a two-step protocol described previously in the literature.[5] Briefly, 0.12 g of fluorographite (*ca.* 4 mmol) was suspended, under inert atmosphere, in 15 mL of N_2-degassed DMF. This suspension was sonicated for 4 h, and then, 1.05 g of KCN (*ca.* 16 mmol) was added. The mixture was reacted at 130 ^oC with magnetic stirring for 24 h. Reactor was cooled down and the black powder was washed by centrifugation with 4x15 mL of fresh DMF, 4x15 mL of water, 4x15 mL of methanol (MeOH) and 4x15 mL of acetone. Drying under vacuum afforded cyano-graphene G-CN. This sample was hydrolysed with a 20% aqueous HNO_3 solution at 100 ^oC for 16 h. It was then washed with water by centrifugation with the necessary amount of cycles till the supernatant reached neutral pH. Drying under vacuum afforded sample GA, which was furtherly used without additional purification.

General procedure for the synthesis of GA-TiO_2 catalyst

A sample of GA (10 mg) was suspended in H_2O (20 mL) by sonicating for 5 min. Then, 8 mg of $Ti(O^iPr)_4$ (0.0275 mmol) was added, and the reaction was allowed to proceed at room temperature for 3h.[6] The afforded black powder was then washed by centrifugation cycles with 3x15 mL of water and 3x15 mL of MeOH. Moreover, once the supernatant of each cycle was discarded and the fresh solvent was added, sample was immediately sonicated for 5 min for a better contaminants removal. Drying under vacuum afforded sample **GA-TiO_2**.

General procedure for the synthesis of compounds 3a-3j

The reaction for the synthesis of 1,3,4-oxadiazoles was performed, if not otherwise stated, as follows: A vial equipped with a magnetic stir bar was charged with acylhydrazide (0.1 mmol), α-keto acid (0.1 mmol), Na_2CO_3 (2 eq.), **GA-TiO_2** (1 mg) and H_2O (1.0 mL, 0.1 M). The reaction mixture was irradiated and stirred in the photoreactor setup using a white LED (see Figure S1) for a desired time, typically 1h, at room temperature and with an intensity of 40 mW cm^{-2}. After reaction was completed, the whole mixture was filtrated over a 0.47 µm polytetrafluoroethylene (PTFE) membrane to separate the catalyst from the organic mixture. The recovered organic phase was extracted with H_2O/EtOAc for three times. The collected organic extracts were dried over $MgSO_4$, filtered and concentrated under vacuum. The crude was purified by flash column chromatography (silica gel) using a mixture of EtOAc/CyH (90:10) to provide the pure product when necessary.

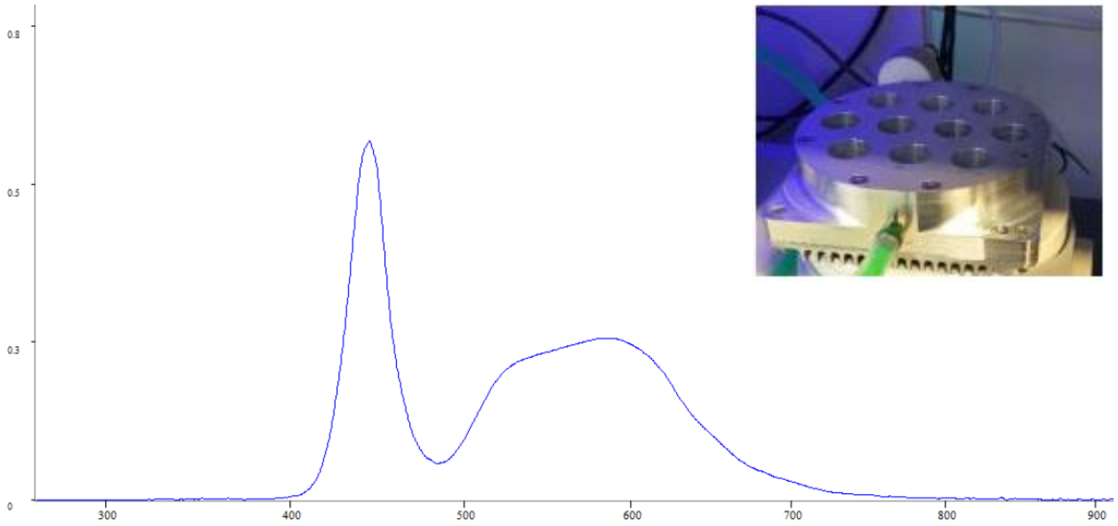

Figure S1. Photoreactor set-up employed and emission spectrum of our white LED.

Organic products characterization

***2,5-diphenyl-1,3,4-oxadiazole* (3a).** Prepared according to the general product to yield a white solid (74 % yield). ^{1}H NMR ($CDCl_3$, 300 MHz): δ 7.86-7.82 (4H, m), 7.50-7.44 (6H, m).[7]

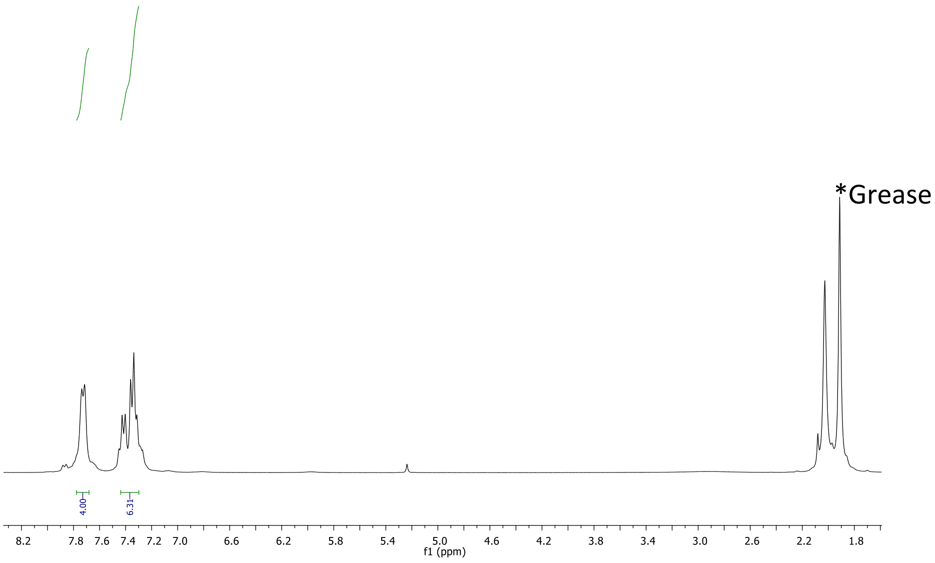

Figure S2. ^{1}H-NMR spectrum of **3a.**

***2,5-di-4-methylphenyl-1,3,4-oxadiazole* (3b).** Prepared according to the general product to yield a white solid (74 % yield). ^{1}H NMR ($CDCl_3$, 300 MHz): δ 7.70 (4H, d, J = 7.4 Hz), 7.33 (4H, d, J = 7.4 Hz), 2.44 (6H, s).[4]

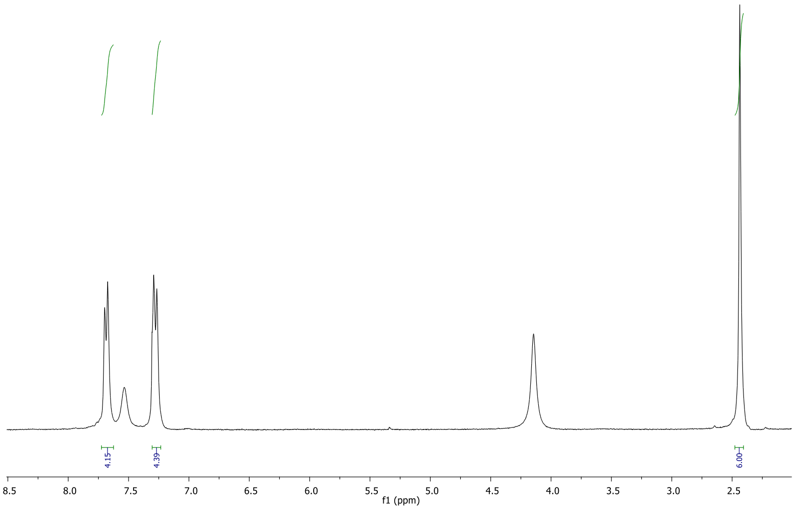

Figure S3. ^{1}H-NMR spectrum of **3b.**

***2-isopropyl- 5-phenyl -1,3,4-oxadiazole* (3c).** Prepared according to the general product to yield a white solid (83 % yield). ^{1}H NMR (DMSO-d_6, 300 MHz): δ 8.0 (2H, m) 7.62-7.53 (2H, m), 3.32-3.24 (1H, m), 1.20 (6H, d, J = 6.8 Hz).[4]

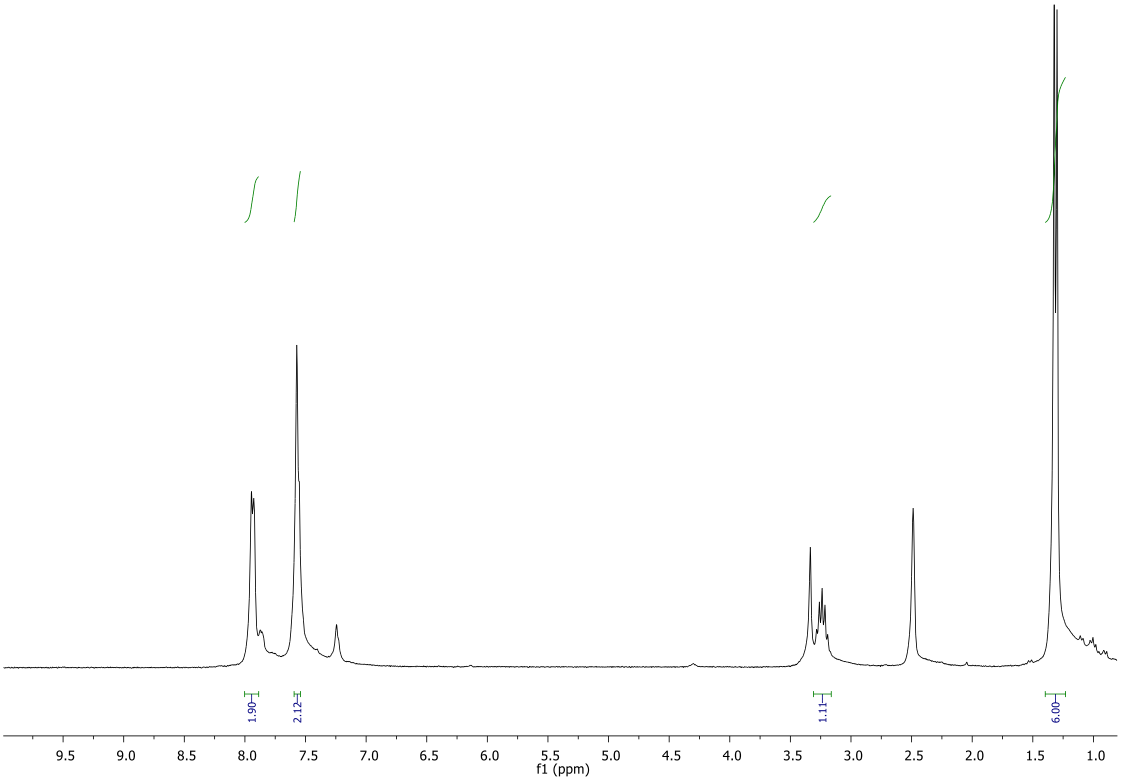

Figure S4. ^{1}H-NMR spectrum of **3c**

***2-phenyl-5-methyl-1,3,4-oxadiazole* (3d).** Prepared according to the general product to yield a white solid (72 % yield). ^{1}H NMR ($CDCl_3$, 300 MHz): δ 7.86 (2H, d, J = 7.7 Hz),7.64 (1H, t, J = 6.9 Hz),7.50 (2H, d, J = 7.7 Hz), 2.26 (3H, s).[8]

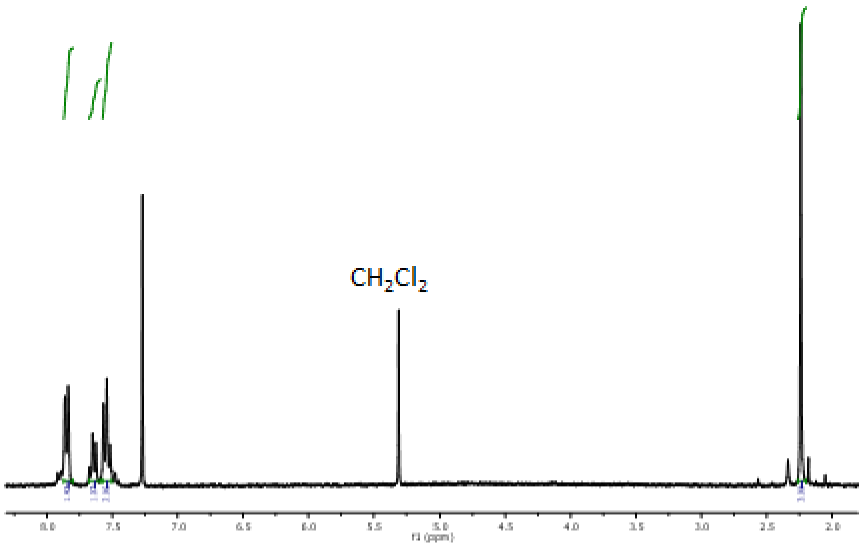

Figure S5. ^{1}H-NMR spectrum of **3d.**

***2-(4-methylphenyl)-5-methyl--1,3,4-oxadiazole* (3e).** Prepared according to the general product to yield a white solid (80 % yield) ^{1}H NMR ($CDCl_3$, 300 MHz): δ 7.77-7.60 (2H, m), 7.23 (2H, d, J = 7.4 Hz), 2.44 (3H, s), 1.96 (3H, s).[5]

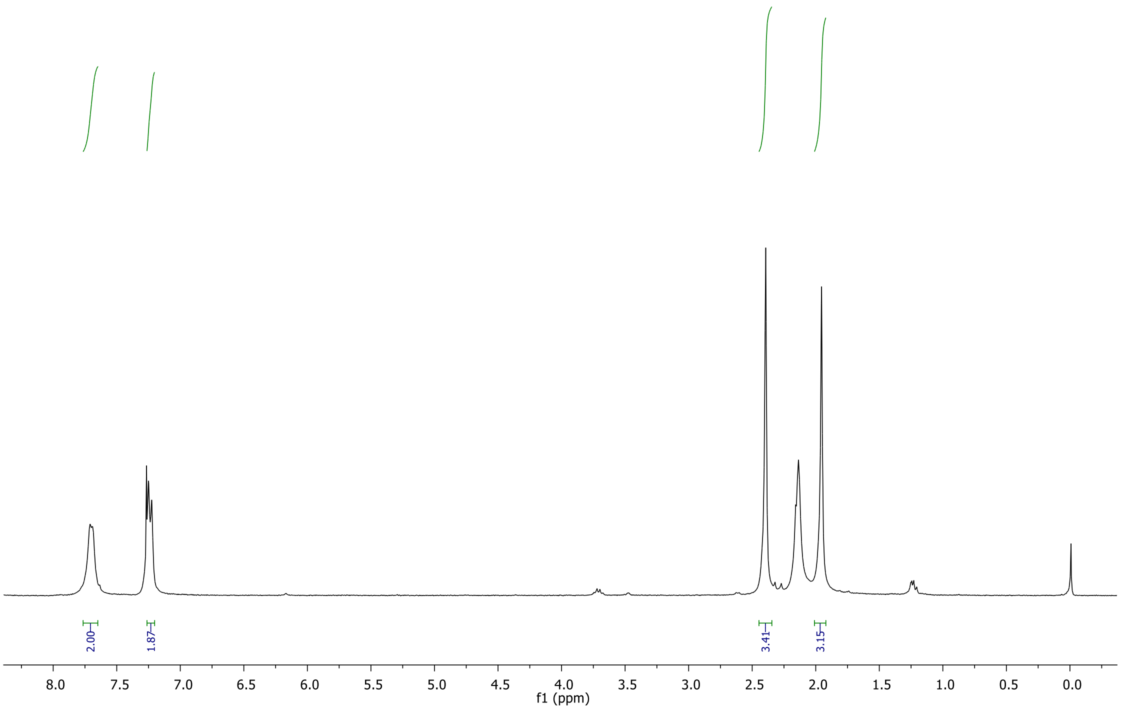

Figure S6. ^{1}H-NMR spectrum of **3e**

***2-(4-(chlorophenyl)-5-methyl 1,3,4-oxadiazole* (3f).** Prepared according to the general product to yield a white solid (70 % yield) ^{1}H NMR ($CDCl_3$, 300 MHz): δ 7.96 (2H, d, J = 7.4 Hz), 7.47 (2H, d, J = 7.4 Hz), 2.62 (3H, s).**[¡Error! Marcador no definido.]**

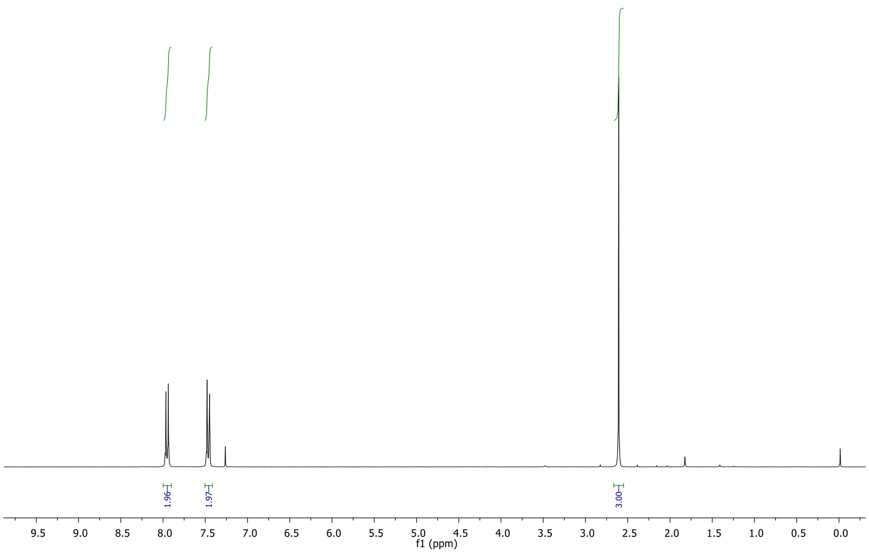

Figure S7. ^{1}H-NMR spectrum of **3f.**

2-(4-(bromophenyl)-5-methyl-1,3,4-oxadiazole **(3g).** Prepared according to the general product to yield a white solid (78 % yield). ^{1}H NMR (CDCl$_3$, 300 MHz): δ 7.79-7.51 (4H, m), 2.06 (3H, s).**¡Error! Marcador no definido.**

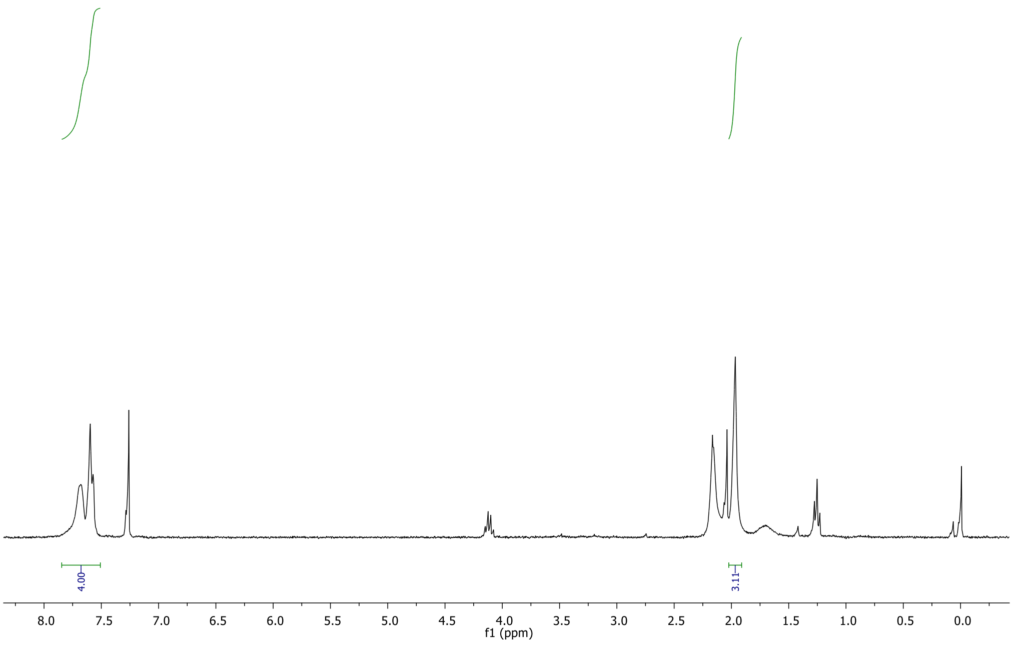

Figure S8. ^{1}H-NMR spectrum of **3g**

2-(4-(trifluoromethyl)phenyl)-5-methyl-1,3,4-oxadiazole **(3h).** Prepared according to the general product to yield a white solid (88 % yield) ^{1}H NMR (CDCl$_3$, 300 MHz): *δ* 8.07-7.96 (2H, m), 7.70 (2H, d, J = 7.4 Hz), 2.02 (3H, s).[9]

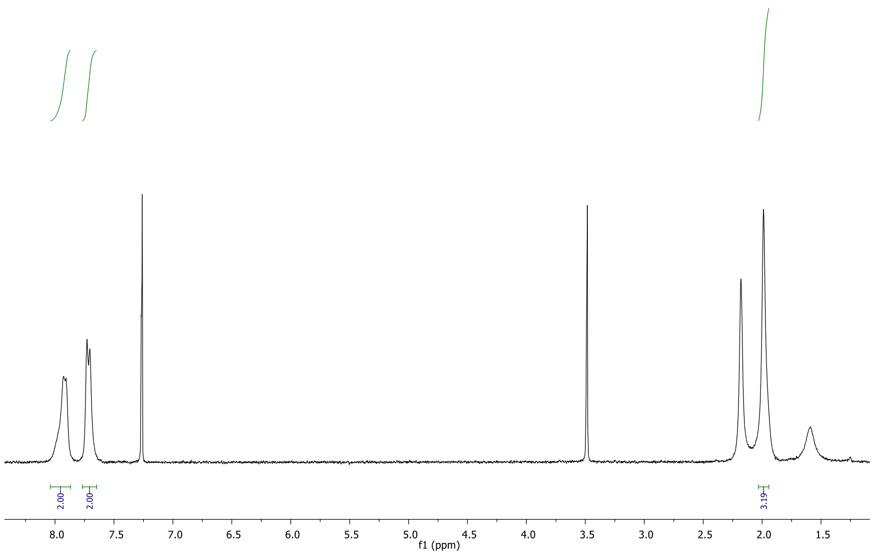

Figure S9. ^{1}H-NMR spectrum of **3h.**

***2-(4-methoxyphenyl)-5-methyl-1,3,4-oxadiazole* (3i).** Prepared according to the general product to yield a white solid (78 % yield) ^{1}H NMR (CD_3OD, 300 MHz): δ 7.81 (2H, d, J = 7.4 Hz), 6.94 (2H, d, J = 7.4 Hz), 3.83 (3H, s), 2.01 (3H, s).[¡Error! Marcador no definido.]

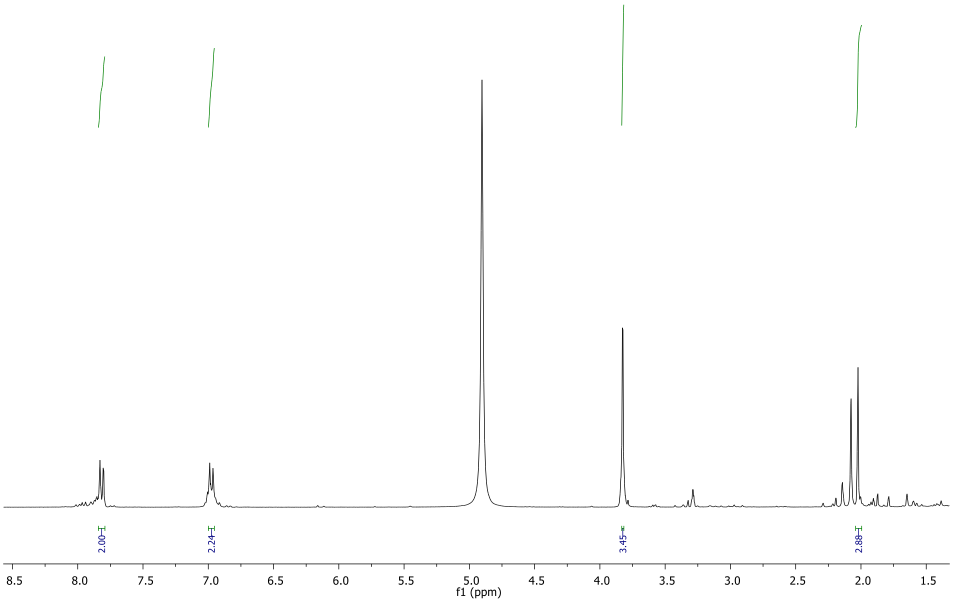

Figure S10. ^{1}H-NMR spectrum of **3i.**

***2-isopropyl-5-methyl -1,3,4-oxadiazole* (3j).** Prepared according to the general product to yield a white solid (74 % yield) ^{1}H NMR (CD_3OD, 300 MHz): δ 2.70 (1H, m), 2.26 (3H, s), 1.20 (6H, d, J=6.8 Hz).[10]

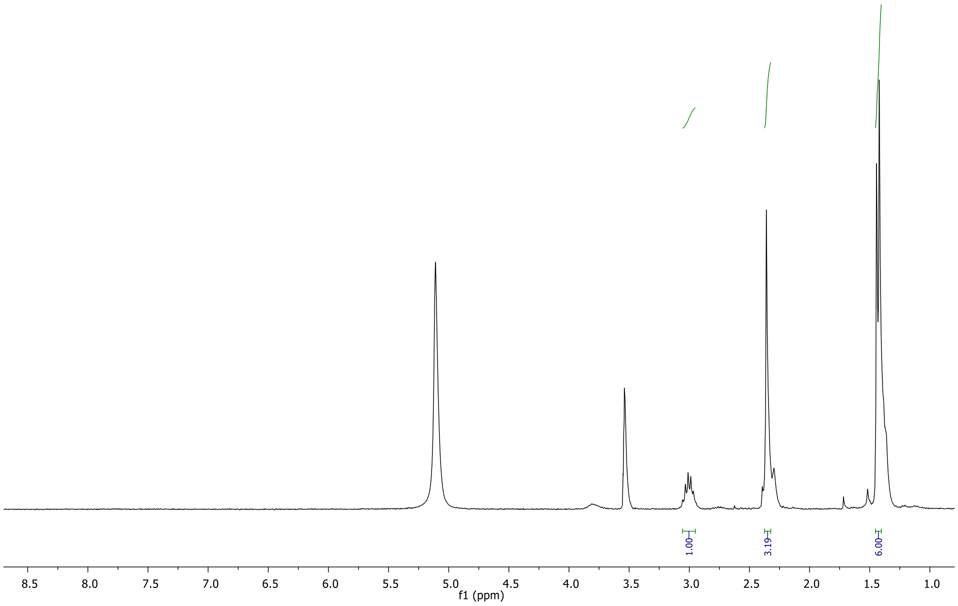

Figure S11. ^{1}H-NMR spectrum of **3j**

S2. SUPPORTING MATERIAL CHARACTERIZATION DATA

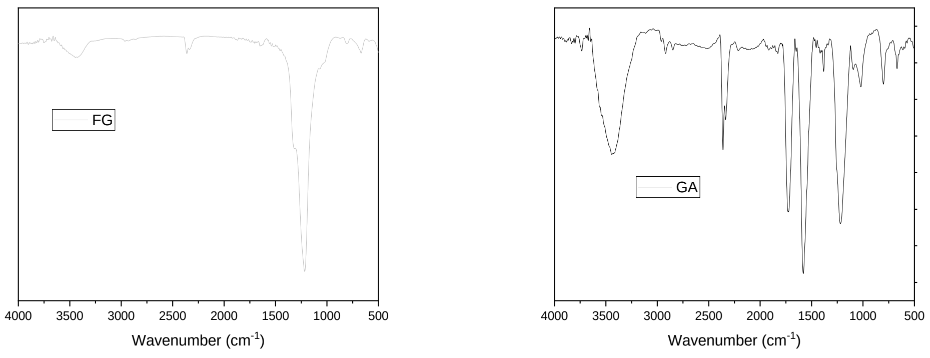

Figure S12. FTIR Spectra of starting fluorographite (FG, left) and freshly produced GA (right).

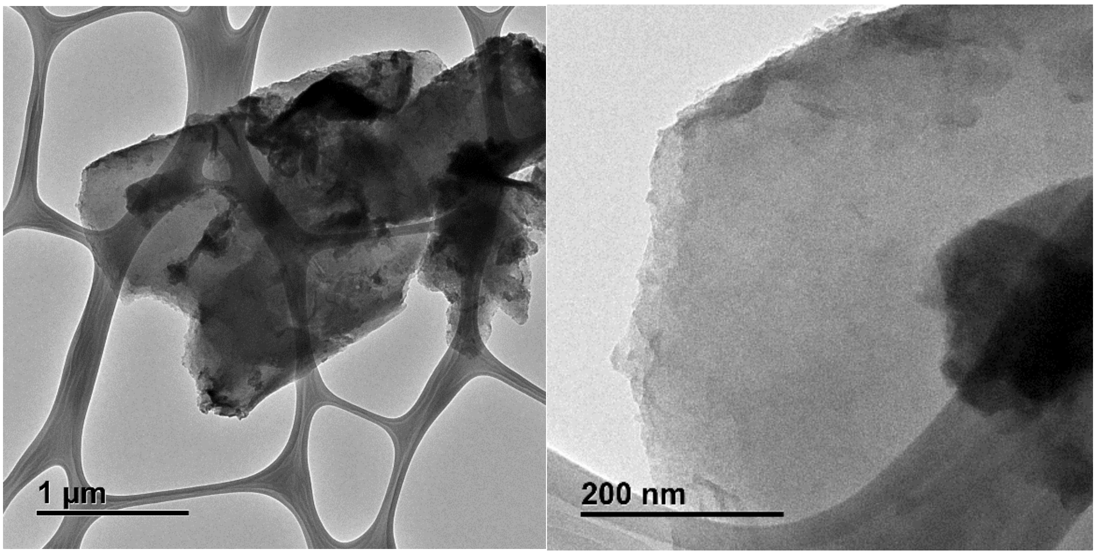

Figure S13. Additional GA TEM images.

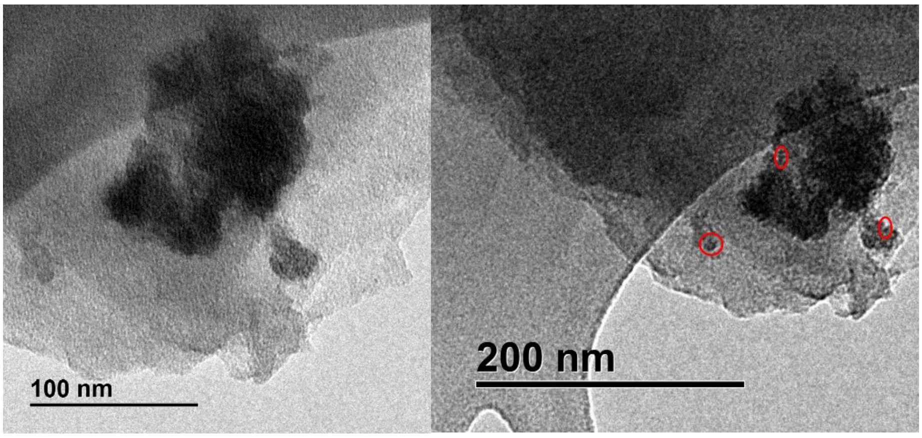

Figure S14. Additional GA-TiO_2 images. Red circles highlight examples of the 4 nm wide nanoparticle.

Table S1. Elemental analysis of samples under study

Sample	C	H	N	O	Ti
GA	65.2	1.9	4.8	28.1	-
GA-TiO_2	50.3	1.8	4.7	37.2	4.4
GA-TiO_2 A.C.	51.2	1.6	4.6	36.8	4.1
Values expressed in %. A.C. denotes recovered after catalysis. The Ti value is determined by TXRF					

Table S2. TXRF analysis of sample **GA-TiO_2**

Element	Line	Conc./ mg/l	Sigma/ mg/l	RSD/ %	LLD/ mg/l	Net area	Backgr.	Chi
Si	K12	135.4	1.4	1.0	1.2	13934	1681	3.00
Cl	K12	Not det.			0.2	78	2454	1.45
K	K12	0.543	0.059	10.9	0.117	737	2788	0.74
Ca	K12	18.67	0.13	0.7	0.10	30720	3266	1.06
Ti	K12	243.78	0.47	0.2	0.05	766358	2833	3.32
Fe	K12	0.212	0.008	3.7	0.012	1617	875	1.24
Co (IS)	K12	10.000	0.041	0.4	0.007	90710	463	3.87
Cu	K12	0.154	0.004	2.7	0.005	2001	376	0.99
Zn	K12	1.004	0.009	0.9	0.004	15273	318	2.06
Br	K12	0.023	0.001	5.5	0.002	566	188	1.48
Sr	K12	0.101	0.002	2.3	0.003	2808	521	1.70

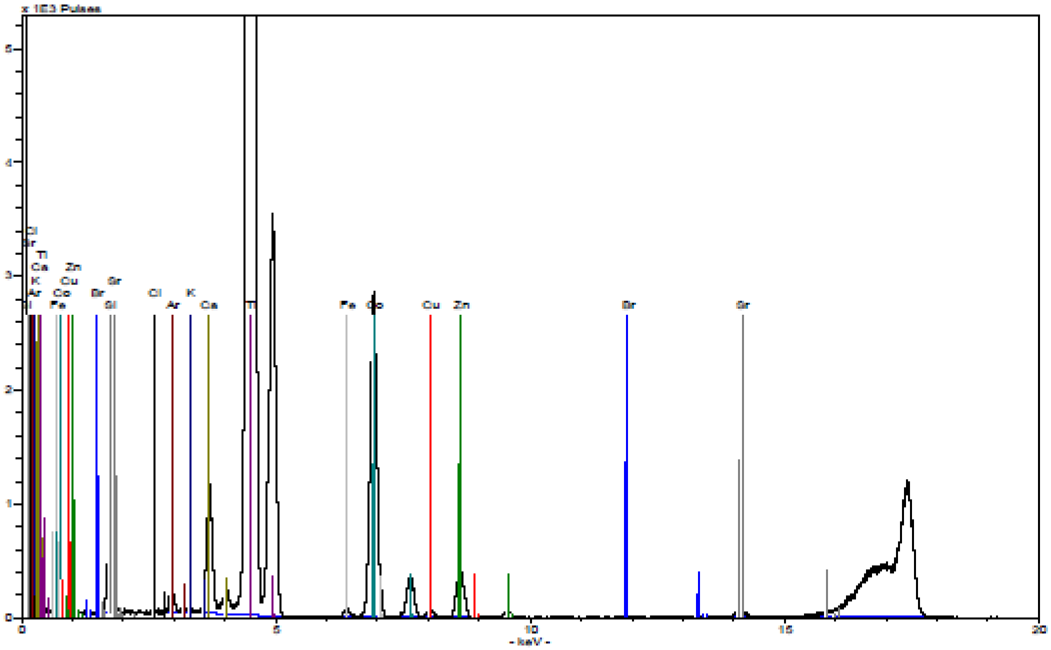

Figure S15. TXRF spectrum of sample **GA-TiO$_2$**

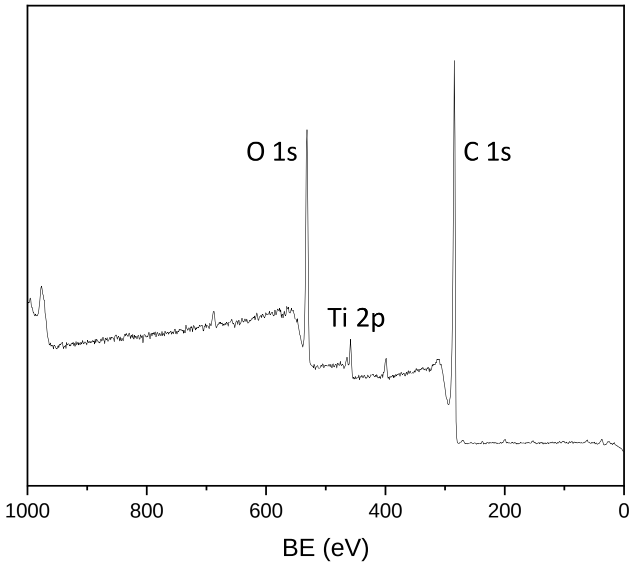

Figure S16. XPS Survey spectrum of sample **GA-TiO$_2$.**

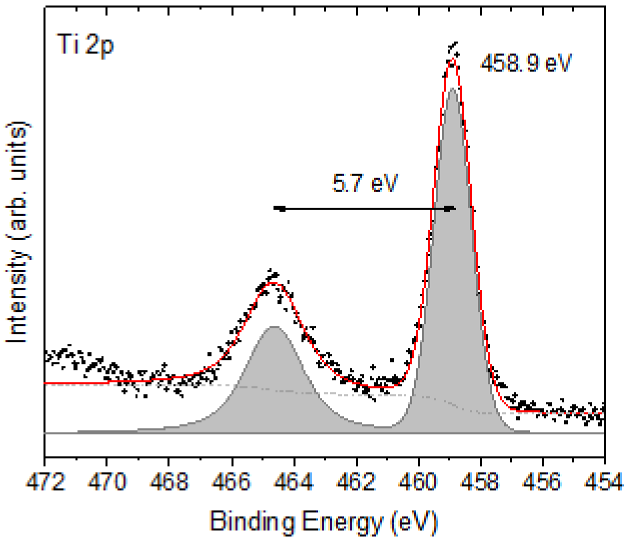

Figure S17. XPS Ti 2p core level region of sample **GA-TiO₂.**

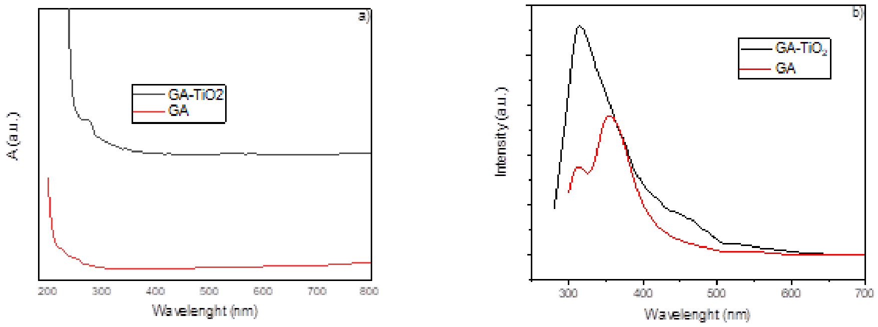

Figure S18. a) UV-Vis absorption and b) emission spectra of samples GA and **GA-TiO₂.**

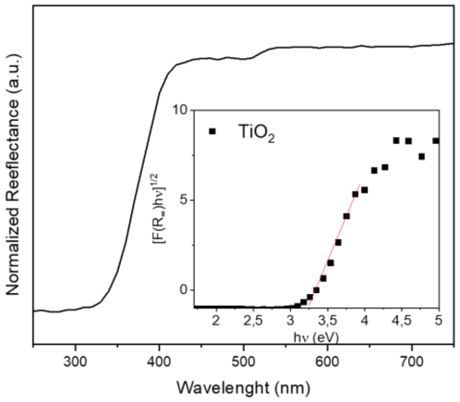

Figure S19. UV-Vis diffuse reflectance spectrum of sample TiO_2. Inset: Tauc plot showing the Kubelka-Munk function with the direct band-gap determination.

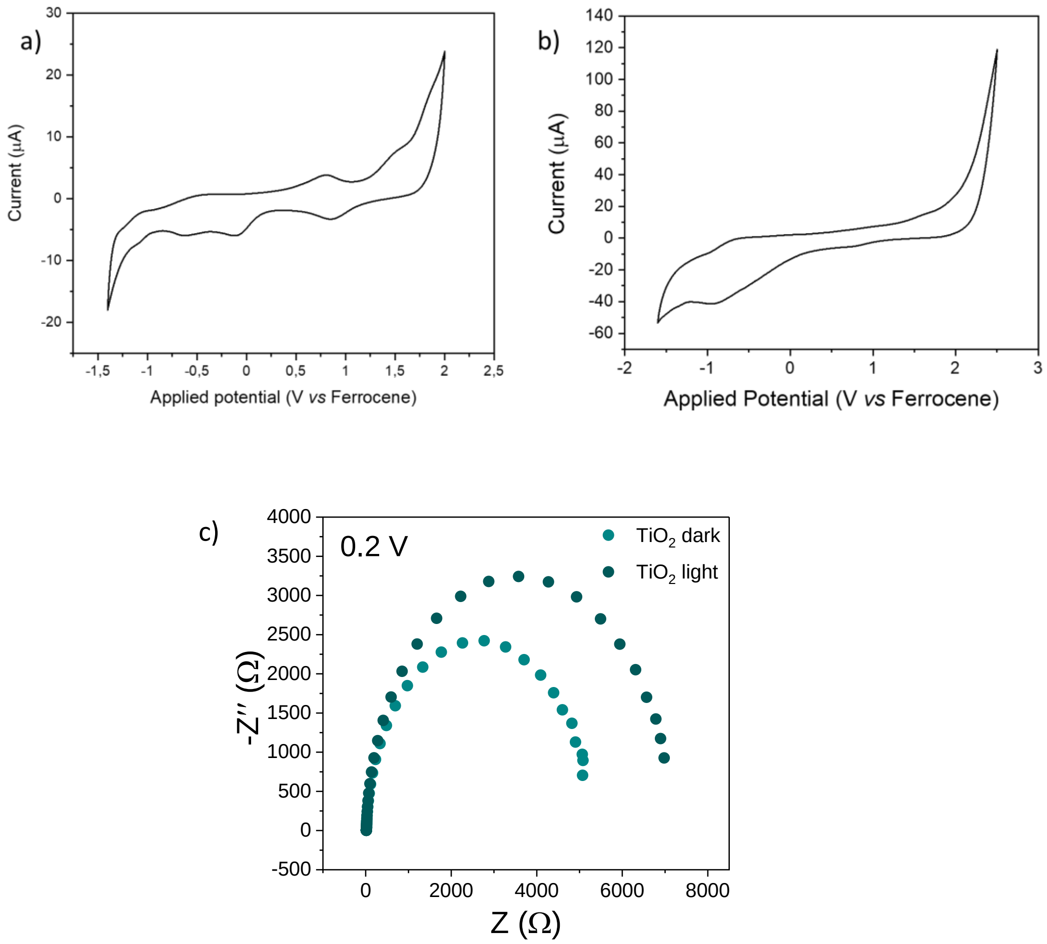

Figure S20. a) CV of TiO_2 (0.1 M NBu_4ClO_4 in DCM); b) CV of **GA-TiO_2** (0.1 M NBu_4ClO_4 in DCM); c) Nyquist plots obtained by Electrochemical Impedance Spectroscopy (EIS) under dark and illumination conditions at 0.2 V vs Ag/AgCl in 0.2 M aqueous Na_2CO_3 of TiO_2 sample.

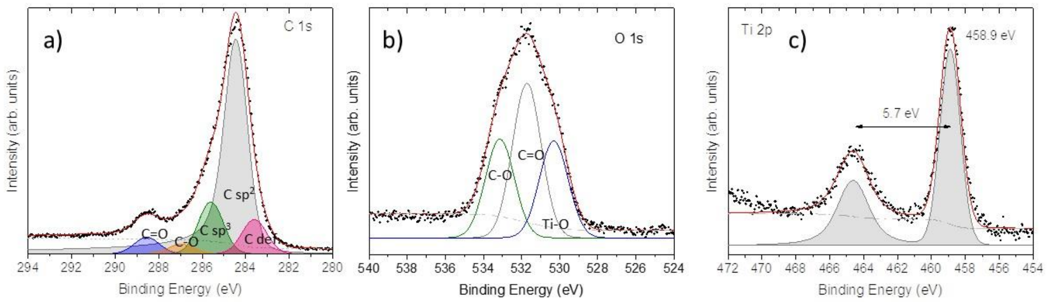

Figure S21. XPS analysis of sample **GA-TiO_2** recovered after the catalytic study. a) C1s b) O1s and c) Ti 2p XPS core level regions.

Table S3. TXRF analysis of sample **GA-TiO_2** recovered after the catalytic study.

Element	Line	Conc./ mg/l	Sigma/ mg/l	RSD/ %	LLD/ mg/l	Net area	Backgr.	Chi
Si	K12	177.5	1.6	0.9	0.8	14544	473	1.69
S	K12	4.50	0.17	3.8	0.24	1353	591	1.13
Cl	K12	Not det.			0.14	39	533	0.68
K	K12	3.978	0.069	1.7	0.061	4304	484	0.83
Ca	K12	10.826	0.099	0.9	0.047	14185	430	1.53
Ti	K12	5.489	0.052	1.0	0.034	13737	808	0.60
V	K12	Not det.			0.019	1	389	0.94
Cr	K12	0.144	0.010	6.8	0.016	567	451	1.01
Mn	K12	0.461	0.012	2.6	0.013	2235	456	1.51
Fe	K12	1.372	0.017	1.2	0.012	8333	594	1.21
Co (IS)	K12	10.000	0.043	0.4	0.007	72215	323	1.93
Ni	K12	0.043	0.004	8.3	0.006	383	313	1.78
Cu	K12	0.097	0.004	4.1	0.005	999	301	0.62
Zn	K12	1.803	0.013	0.7	0.006	21822	541	2.00
Br	K12	0.049	0.002	3.8	0.002	941	148	3.14
Sr	K12	0.053	0.002	3.7	0.003	1164	328	4.46

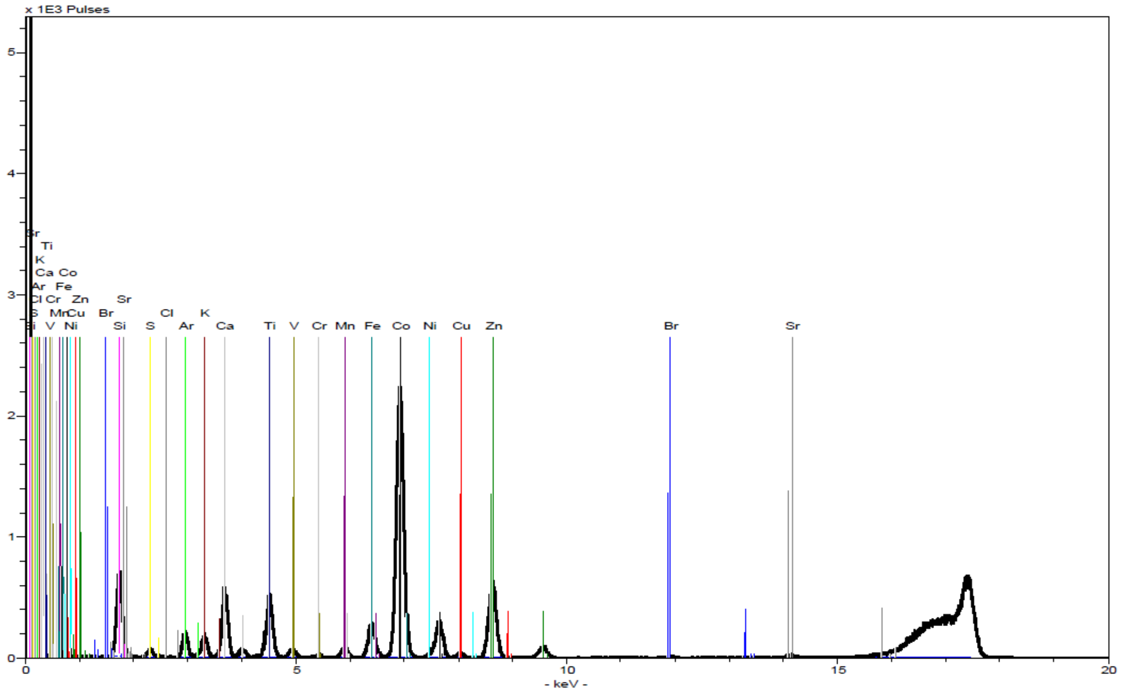

Figure S22. TXRF spectrum of sample **GA-TiO_2** recovered after the catalytic study.

S3. SUPPORTING CATALYTIC DATA

Table S4. Optimization of the catalytic reaction conditions for the light-driven synthesis of 1,3,4-oxadiazoles.

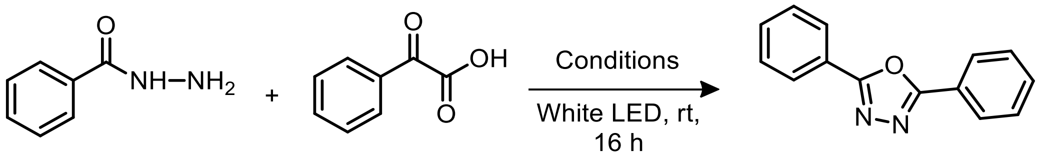

Catalyst	Base	Solvent	Conversion oxadiaz. (%)
No	K_2CO_3	DMF	0
GA-TiO_2 (inert atm)	K_2CO_3	DMF	0
GA-TiO_2	K_2CO_3	DMF	14
GA-TiO_2	DIPEA	DMF	65
GA-TiO_2	Et_3N	DMF	64
GA-TiO_2	Bu_3N	DMF	0
GA-TiO_2	NaOH	DMF	0
GA-TiO_2	DIPEA	MeCN	0
GA-TiO_2	DIPEA	DCM	0
GA-TiO_2	DIPEA	MeOH	30
GA-TiO_2	DIPEA	DMSO	0
GA-TiO_2	DIPEA	H_2O	64
GA-TiO_2	Et_3N	H_2O	40
GA-TiO_2	NaOH	H_2O	4
GA-TiO_2	K_2CO_3	H_2O	17
GA-TiO_2	Na_2CO_3	H_2O	>98

Reaction conditions: 0.1 mmol of each substrate, 2 equivalent of base, 1 mL of solvent, 1 mg of GA-TiO_2 as catalyst, under air atmosphere for 16 h at room temperature. Conversions determined by ^{1}H-NMR

Table S5. Light screening in the synthesis of 1,3,4-oxadiazoles.

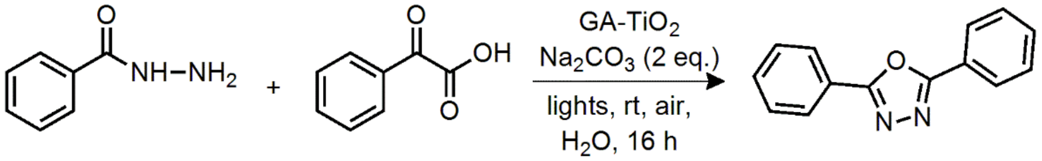

Light (Power)	Conversion oxadiazole (%)
No	0
360 nm (10 mW cm^{-2})	Decomposition product
385 nm (8 mW cm^{-2})	38
420 nm (18 mW cm^{-2})	12
450 nm (22 mW cm^{-2})	3
520 nm (22 mW cm^{-2})	0
White (40 mW cm^{-2})	>98

Reaction conditions: 0.1 mmol of each substrate, 2 equivalent of base, 1 mL of solvent, 1 mg of **GA-TiO₂** as catalyst, under air atmosphere for 16 h at room temperature. Conversions determined by ^{1}H-NMR

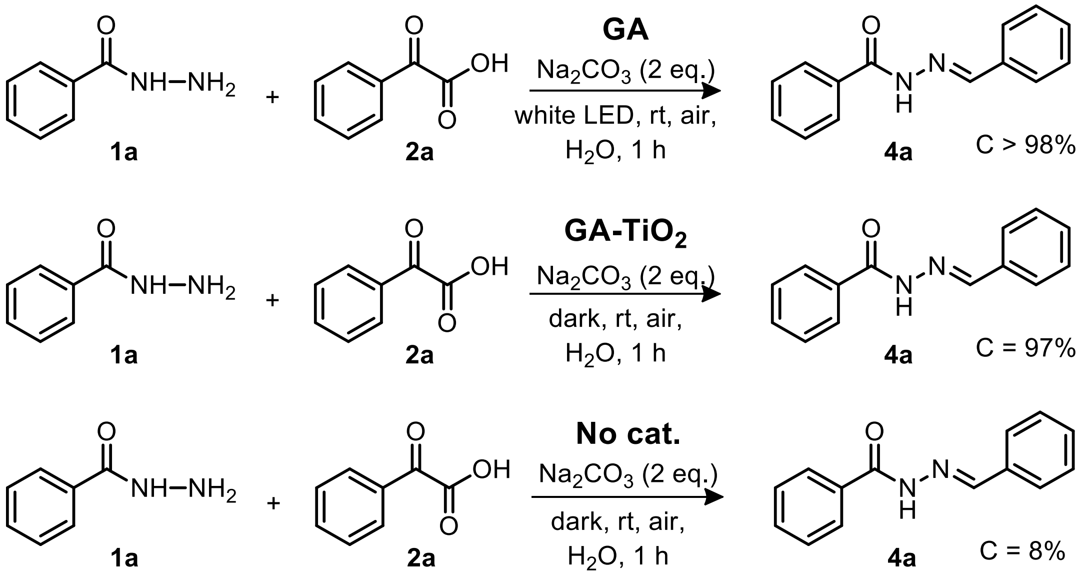

Scheme S1. Control experiments with GA, **GA-TiO$_2$** and non-catalyzed reaction for mechanistic elucidation. Note that not further evolution of the imine towards the oxadiazole was observed.

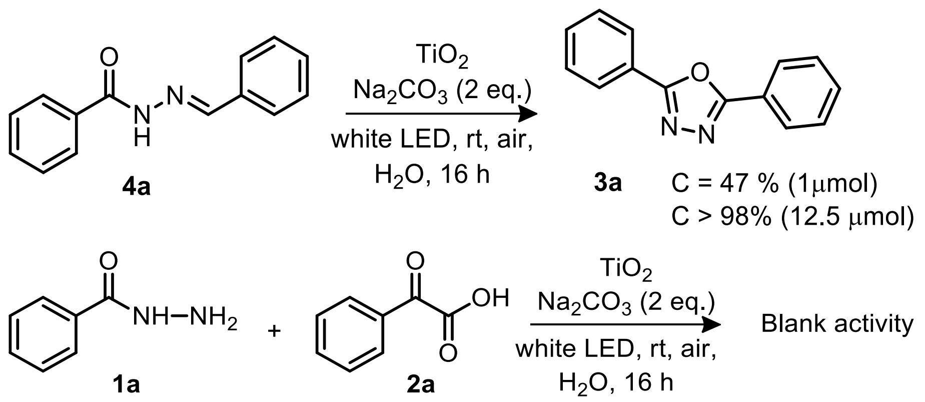

Scheme S2. Control experiments with TiO_2 for mechanistic elucidation.

Table S6. Comparison in the activity of **GA-TiO_2** with other state-of-the-art catalysts

Catalyst	Substrate	Loading	Time	Vector	Ref
GA-TiO_2	Ketoacid + hydrazide	2 mmol vs 1 mg	1h	White LED	This work
GO	Benzonitrile one pot aminooxime and aldehyde	1 mmol vs 25 mg	24 h	80 °C	11
GO-MnO_x	Aldehyde + aminooxime	1 mmol vs 140 mg	24 h	95 °C	12
[Pd]	Aminooxime + aryl iodide + CO	5 mol%	15 h	95 °C	13
[Pd]	Oxadiazole + amine	10 mol%	12 h	120 °C	14
[Pd]	Tetrazole + $CHCl_3$ + Aryl iodide	10 mol%	24 h	120 °C	15
PANI-C_3N_4-TiO_2	Ketoacid + hydrazide	0.5 mmol vs 40 mg	24 h	White LED	16
S_8 + K-PHI	Imine	0.06 mmol vs 5 mg	20 h	Blue LED	17
Carbazole	Hypervalent iodide-diazonium salt	5 mol%	5 h	White LED	18
Fe-MOF	Tetrazole	1 mmol vs 5 mol% (Fe)	2 h	25 °C	19
Al-K-Clay	Hydrazide	100 mg vs 75 mg	15 min	55 °C MW	20
$HClO_4$-SiO_2	Aminooxime + anhydride	5 mol%	5 min	80 °C	21

HAP-SO_3H	Aminooxime + aldehyde, I_2	20 % wt.	24 h	80 °C	22

Table S7. TXRF analysis of the reaction liquid in a reaction employing **GA-TiO_2**

Element	Line	Conc./ mg/l	Sigma/ mg/l	RSD/ %	LLD/ mg/l	Net area	Backgr.	Chi
Si	K12	244.4	2.7	1.1	1.7	12896	917	1.30
Cl	K12	302.9	1.5	0.5	0.4	96581	1432	6.74
K	K12	3.176	0.089	2.8	0.104	2214	582	2.36
Ca	K12	6.62	0.11	1.6	0.08	5584	492	1.17
Ti	K12	0.292	0.021	7.3	0.034	471	328	1.87
Fe	K12	Not det.			0.015	15	369	2.65
Co (IS)	K12	5.000	0.042	0.8	0.011	23256	311	0.97
Ni	K12	Not det.			0.010	5	363	0.95
Cu	K12	0.025	0.004	16.6	0.008	165	284	1.67
Zn	K12	0.013	0.003	24.4	0.006	97	231	2.21
Br	K12	0.597	0.008	1.4	0.004	7383	297	1.06

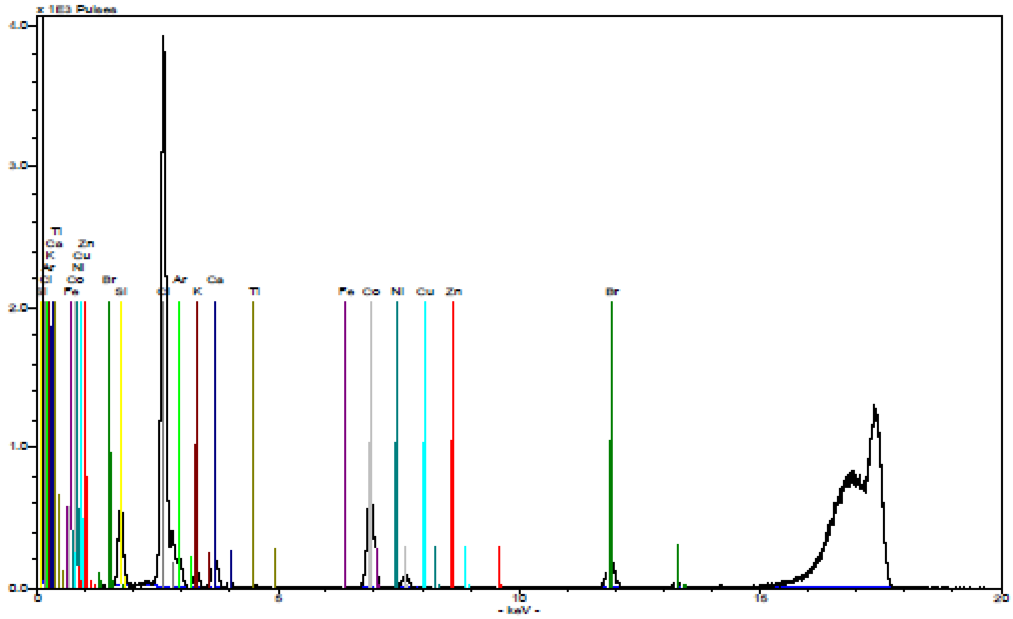

Figure S23. TXRF spectrum of the reaction liquid in a reaction employing GA-TiO_2.

S4. SUPPORTING REFERENCES

[1] R: Yabe, Y. Ebe, T. Nishimura. *Chem. Commun.* **2021**, *57*, 5917-5920.

[2] C. G. López-Calixto, M. Barawi, M. Gomez-Mendoza, F. E. Oropeza, F. Fresno, M. Liras, V. A. de la Peña O'Shea. *ACS Catal.* **2020**, *10*, 9804-9812.

[3] D. Mosconi, M. Blanco, T. Gatti, L. Calvillo, M. Otyepka, A. Bakandritsos, E. Menna, S. Agnoli, G. Granozzi. *Carbon* **2019**, *143*, 318-328.

[4] M. Luna, M. Barawi, S Gómez-Moñivas, J. Colchero, M. Rodríguez-Peña, S. Yang, X. Zhao, Y.-H. Lu, R. Chintala, P. Reñones, V. Altoe, L. Martínez, Y. Huttel, S. Kawasaki, A. Weber-Bargioni, V. A. de la Peña O´Shea, P. Yang, P. D. Ashby, M. Salmeron. *ACS Appl. Mater. Interfaces* **2021**, *13*, 50531-50538.

[5] A. Bakandritsos, M. Pykal, P. Błoński, P. Jakubec, D. D. Chronopoulos, K. Poláková, V. Georgakilas, K. Čépe, O. Tomanec, V. Ranc, A. B. Bourlinos, R. Zbořil and M. Otyepka, *ACS Nano*, **2017**, *11*, 2982–2991.

[6] H. Li, X. Cui. *Int J. Hydrogen Ener.* **2014**, *39*, 19877-19886

[7] K. R. Paidi, M. K. Kolli, E. K. Reddy, V. R. Pedakotla. *Chemistry of Heterocyclic Compounds* **2020**, *56*, 371–376.

[8] Q. Li, Y. Tao, D. Xu, H. Zhang, L. Duan. *J. Chin. Chem. Soc.* **2014**, *61*, 665-670.

[9] a) K. K. Gnanasekaran, B. Nammalwar, M. Murie, R. A. Bunce. *Tetrahedron Lett.* **2014**, *50*, 6776-6778; b) L. A. Trimble, M. W. Sumarah, B. A. Blackwell, M. D. Wrona, J. D. Miller. *Tetrahedron Lett.*, **2012**, *53*, 956-958.

[10] J. Löffler, R. Schobert. *Synlett* **1997**, *3*, 283-284.

[11] P. Basak, S. Dey, P. Ghosh. *RSC Adv.* **2021**, *11*, 32106-32118

[12] F. Saadati, B. Kaboudin, R. Hasanloei, Z. Namazifar, X. Marset, G. Guillena. *Appl. Organometallic Chem.* **2020**, *34*, e5838.

[13] J. R. Young, R. J. DeVita. *Tetrahedron Lett.* **1998**, *39*, 3931-3934

[14] Y. B. Bhujabal, K. S. Vadagaonkar, A. R. Kapdi. *Asian J. Org. Chem.* **2019**, *8*, 289-295.

[15] Z. Li, L. Wang. *Adv. Synth. Catal.* **2015**, *357*, 3469-3473.

[16] L. Wang, Y. Wang, Q. Chen, M. He. *Tetrahedron Lett.* **2018**, *59*, 1489-1492.

[17] B. Kurpil, K. Otte, M. Antonietti, A. Savateev. *Appl. Catal. B* **2018**, *228*, 97-102.

[18] J. Li, X.-C. Lu, Y. Xu, J.-X. Wen, G.-Q. Hou, L. Liu. *Org. Lett.* **2020**, *22*, 9621-9626

[19] S. Goswami, H. S. Jena, S. Konar. *Inorg. Chem.* **2014**, *53*, 7071-7073

[20] D. Suresh, K. Kanagaraj, K. Pitchumani. *Tetrahedron Lett*. **2014**, *55*, 3678-3682.

[21] R. Tadikonda, M. Nakka, M. B. Gajula, S. Rayavarapu, P. R. Gollamudi, S. Vidavalur. *Synthetic Commun.* **2014**, *44*, 1978–1986.

[22] A.-E. El Mansouri, A. Oubell, M. Maatallah, M. Y. AitItto, M. Zahouily, H. Morjani. H. B.Lazrek. *Bioorganic¬Medicinal Chem. Lett*. **2020**, *30*, 127438.
